# The Lambeosaurine Dinosaur *Magnapaulia laticaudus* from the Late Cretaceous of Baja California, Northwestern Mexico

**DOI:** 10.1371/journal.pone.0038207

**Published:** 2012-06-12

**Authors:** Albert Prieto-Márquez, Luis M. Chiappe, Shantanu H. Joshi

**Affiliations:** 1 Bayerische Staatssammlung für Paläontologie und Geologie, Munich, Germany; 2 The Dinosaur Institute, Natural History Museum of Los Angeles County, Los Angeles, California, United States of America; 3 Laboratory of Neuro Imaging, Department of Neurology, UCLA School of Medicine, Los Angeles, California, United States of America; University of Pennsylvania, United States of America

## Abstract

The taxonomy, osteology, phylogenetic position, and historical biogeography of the lambeosaurine hadrosaurid *Magnapaulia laticaudus* (new combination) are revised. The diagnosis of this species is amended on the basis on two autapomorphies (i.e., longest haemal arches of proximal caudal vertebrae being at least four times longer than the height of their respective centra; base of prezygapophyses in caudal vertebrae merging to form a bowl-shaped surface) and a unique combination of characters (i.e., downturned cranioventral process of the maxilla; tear-shaped external naris with length/width ratio between 1.85 and 2.85; neural spines of dorsal, sacral, and proximal caudal vertebrae being at least four times the height of their respective centra). A maximum parsimony analysis supports a sister taxon relationship between *M. laticaudus* and *Velafrons coahuilensis*. Both taxa constitute a clade of southern North American lambeosaurines, which forms a sister relationship with the diverse clade of helmet-crested lambeosaurines from northern North America that includes well-known genera like *Corythosaurus*, *Lambeosaurus*, and *Hypacrosaurus*. According to the results of a Dispersal-Vicariance analysis, southern North American lambeosaurines split from the northern forms via vicariance from a common ancestor that lived in both the northern and southern regions of the continent.

## Introduction

Lambeosaurines form a major clade of “duck-billed” (Hadrosauridae) dinosaurs that are remarkable for their bewildering variety of hollow supracranial crests [1], [2]. These crests enclose hypertrophied nasal passages that have apomorphically migrated caudodorsally relative to the ancestral antorbital position [3],[4]. The lambeosaurine fossil record is widespread in Europe, Asia, and the Americas, spanning the Santonian through the late Maastrichtian stages of the Late Cretaceous [5], [6]. However, approximately half of the more than 20 species currently recognized have been found in North America, most of them having been recovered from late Campanian to early Maastrichtian strata of the northern sedimentary basins of the continent [6]. So far, the lambeosaurine taxa known from southern North America consist of two species from Mexico, *“Lambeosaurus” laticaudus* from the El Gallo Formation of Baja California Norte [7] and *Velafrons coahuilensis* from the Cerro del Pueblo Formation of Coahuila [8], and four forms from the USA, *Angulomastacator daviesi* from the Aguja Formation of Texas [9], *Parasaurolophus cyrtocristatus* from the Fruitland or lower Kirtland Formation of New Mexico [10] and the Kaiparowits Formation of Utah [11], *P. tubicen* from the Kirtland Formation of New Mexico [10], and an indeterminate lambeosaurine from the Ojo Alamo Formation of New Mexico [12]. No species of hollow crested hadrosaurid has been named in South America, although various appendicular and axial hadrosaurid remains from Argentina have been referred to Lambeosaurinae indeterminate [13]–[15].

**Figure 1:**
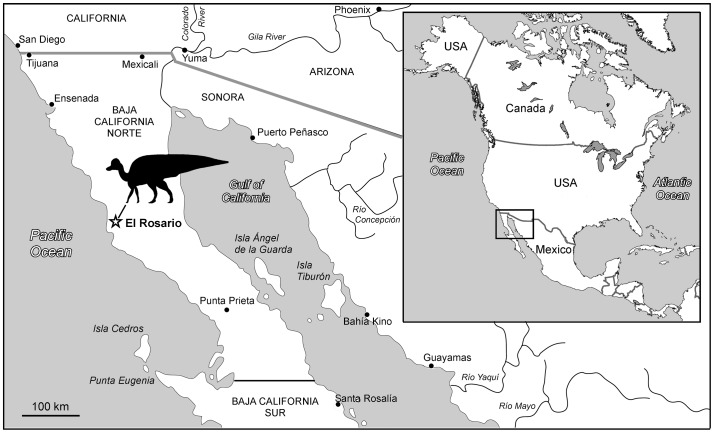
Geographical setting of *Magnapaulia laticaudus*. Map of Baja California Norte showing the location of El Rosario; the remains of *M. laticaudus* were found no more than three miles west of El Rosario.

Although they are rare in the Pacific coast of North America, dinosaurian and other vertebrate fossils are comparatively common in the late Campanian deposits of the El Gallo and La Bocana Roja formations outcropping in the vicinity of the town of El Rosario (Fig. 1), north and south of the Arroyo del Rosario creek, near the coastline of southern Baja California Norte, northwestern México [16]–[21]. Such remains document a diverse Cretaceous fauna composed of amphibians, lizards, turtles, mammals, crocodilians, and ornithischian and saurischian dinosaurs, including birds [17]–[20], [22]–[24]. The fossils of hadrosaurids, however, are the most abundant in those strata.

Morris [16], [25], [26] documented the discovery of a few cranial and numerous postcranial bones that he recognized as those of a large form of lambeosaurine hadrosaurid. Morris [16] originally referred these hadrosaurian remains to *Hypacrosaurus altispinus* based on the long neural spines of the caudal vertebrae and the boot-shaped distal process of the ischium. Later, however, this author amended his referral to cf. *Lambeosaurus* based on the relatively narrow and elongate morphology of the external naris in the premaxilla of this animal, which more closely resembles that of *Lambeosaurus* than the shorter and broader naris of *H. altispinus* [25]–[27]. Finally, Morris [7] erected a new species for the lambeosaurine of Baja California Norte, *L. laticaudus*. He provided a differential diagnosis in which this taxon is differentiated from other species of *Lambeosaurus* by having a deep tail characterized by caudal vertebrae with elongate haemal arches that match the great length of the neural spines. Additionally, Morris [7] included the large size reached by some individuals of *L. laticaudus* (estimated in 14–15 m in length) as diagnostic for this species.

Although in his original work Morris [7] provided a brief description and line drawings of the cranial materials, the appendicular and axial skeleton remained largely undescribed. Here, we present a detailed osteological description of the cranial, axial, and appendicular skeletal remains available for this hadrosaurid, updating its comparative anatomy in the context of the increasing diversity of lambeosaurine species discovered in the three decades since Morris' original studies. In doing so we revise the taxonomy of this lambeosaurine, showing that it merits placement in a new genus. Furthermore, cladistic and biogeographic numerical analyses are conducted for inferring the phylogenetic position of this species within Lambeosaurinae and its biogeographical implications for the clade, with emphasis in elucidating when and under which mechanisms did southern North American lambeosaurines achieve their recorded distribution. Ultimately, this study fills a gap in our knowledge of one of the less understood chapters in the evolutionary history of hadrosaurids: the diversity and evolution of the lambeosaurine dinosaurs from the Pacific coast and southern North America.

### Institutional abbreviations

AEHM, Amur Natural History Museum, Blagoveschensk, Russia; AMNH, American Museum of Natural History, New York, U.S.A.; CMN, Canadian Museum of Nature, Ottawa, Canada; FMNH, The Field Museum, Chicago, U.S.A.; GMV, National Geological Museum of China, Beijing, China; IVPP, Institute of Vertebrate Paleontology and Paleoanthropology, Beijing, China; LACM, Natural History Museum of Los Angeles County, Los Angeles, U.S.A.; MOR, Museum of the Rockies, Bozeman, Montana, U.S.A.; MSNM, Museo Civico di Storia Naturale di Milano, Milan, Italy; NHM, The Natural History Museum, London, U.K.; NMMNH, New Mexico Museum of Natural History and Science, Albuquerque, U.S.A.; ROM, Royal Ontario Museum, Toronto, Ontario, Canada; SM, Senckenberg Museum, Frankfurt am Main, Germany; SDNHM, San Diego Natural History Museum, San Diego, U.S.A.; TMP, Royal Tyrrell Museum of Paleontology, Drumheller, Canada; UALVP, University of Alberta, Laboratory of Paleontology, Edmonton, Canada; YPM, Yale Peabody Museum of Paleontology, New Haven, U.S.A.; ZPAL, Institute of Paleobiology, Polish Academy of Sciences, Warsaw, Poland.

## Results

### Systematic Paleontology

Dinosauria Owen, 1842 [28]

Ornithischia Seeley, 1887 [29]

Ornithopoda Marsh, 1881 [30]

Hadrosauridae Cope, 1870 [31]

Lambeosaurinae Parks, 1923 [32]

*Magnapaulia* gen. nov.

#### Etymology

*Magna*, the Latin for “large”, refers to the unusually large size reached by at least some specimens of this lambeosaurine; *paulia* honors Mr. Paul Haaga for his outstanding support to the research and public programs of the Natural History Museum of Los Angeles County and its Dinosaur Institute.

#### Diagnosis

As for the only known species.

*Magnapaulia laticaudus* Morris, 1981 [7] new combination

urn:lsid:zoobank.org:act:FD9EDA56-7E2C-4F7D-A567-3BB1C4360C8E

Figs. 2–21

**Figure 2:**
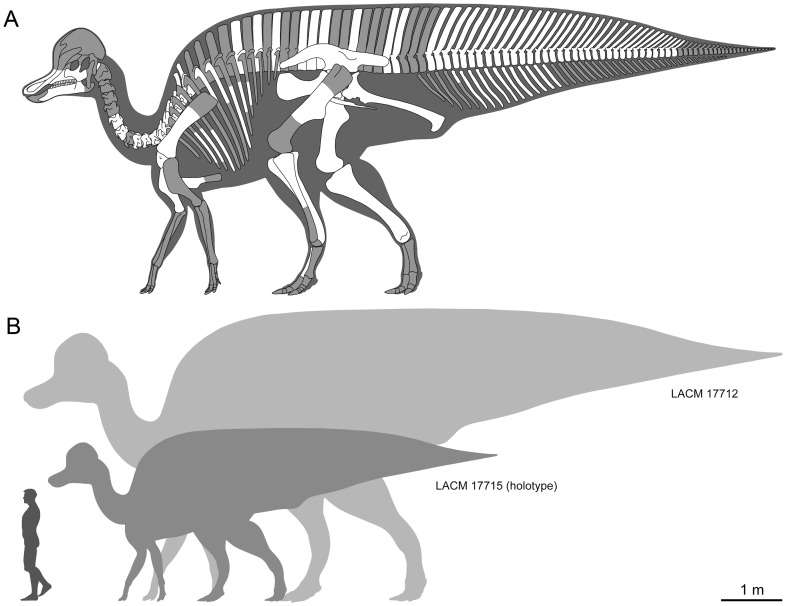
Summary of skeletal fossil representation and size range of *Magnapaulia laticaudus*. Idealized lambeosaurine skeleton showing the elements preserved in *T. laticaudus* (cranial crest, forelimbs, and pedes of the model are based on *Corythosaurus casuarius* skeletons AMNH 5240 and 5338) (A). Size range of known *M. laticaudus* specimens (B).

**Figure 3:**
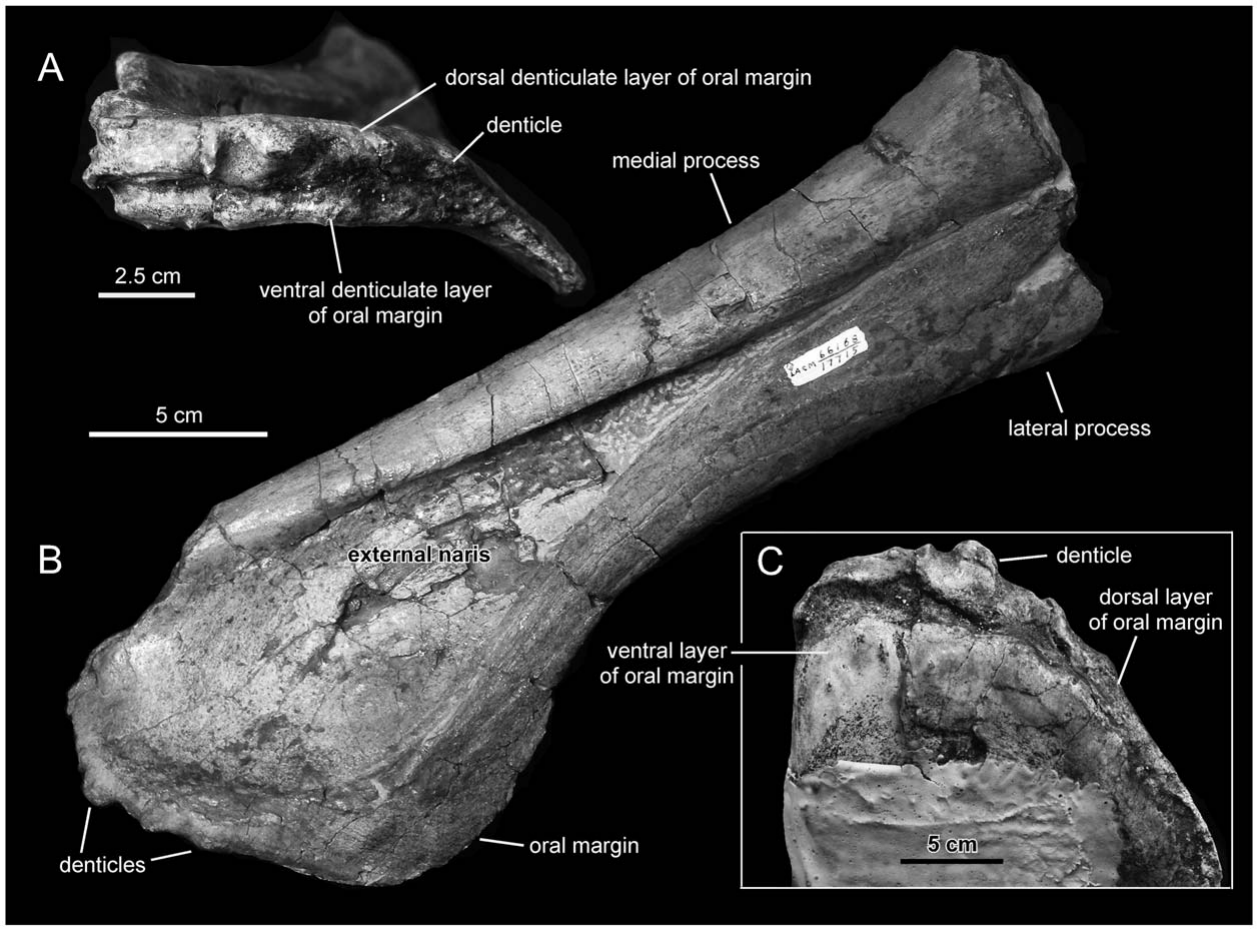
*Magnapaulia laticaudus*, LACM 17715 (holotype), left premaxilla. Premaxilla in rostral and slightly lateral (A), dorsolateral view (B), and ventral (C) views.

**Figure 4:**
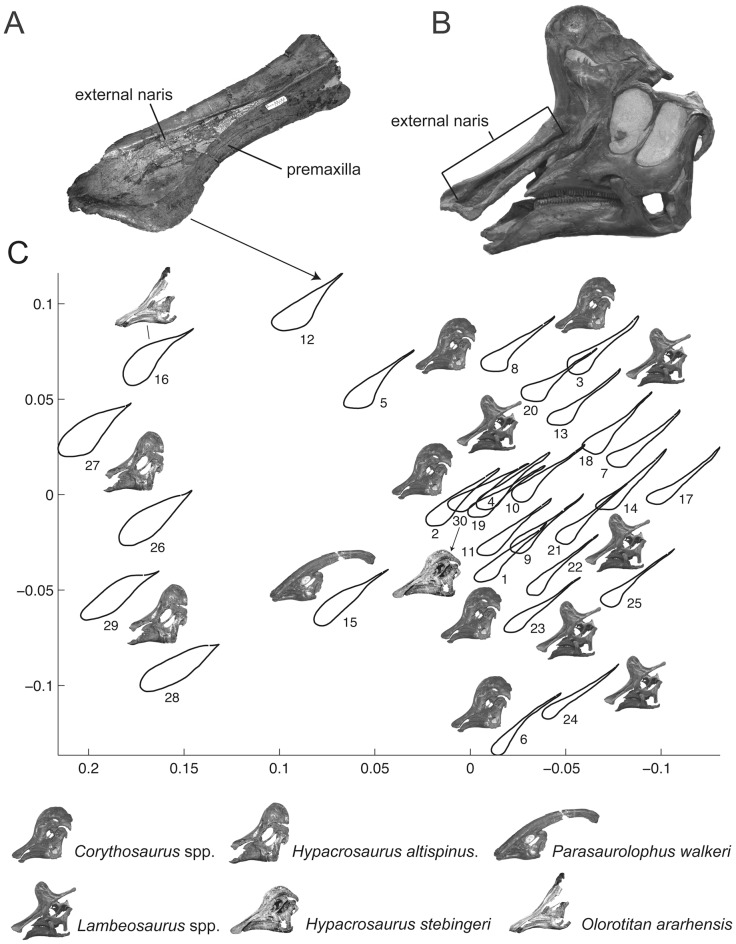
Square-Root Elastic morphometric analysis [**33]**, [**34**] of a set of boundaries corresponding to the external naris of various species of lambeosaurine dinosaurs. Left premaxilla of *Magnapaulia laticaudus* (LACM 17715) in lateral view (A). Left lateral view of a skull of *Lambeosaurus clavinitialis* (CMN 8703), showing the location of the external naris (B). Bivariate plot of the two first axes of the Non-metric Multidimensional Scaling analysis performed on the results of the morphometric analysis (C). The shapes included in the analysis correspond to the following specimens: 1. *Corythosaurus intermedius* (CMN 8676). 2. *C. casuarius* (ROM 1933). 3. *C. casuarius* (TMP 84.121.1). 4. *C. casuarius* (ROM 868). 5. *C. casuarius* (AMNH 5338). 6. *C. intermedius* (ROM 845). 7. *C. intermedius* (CMN 8503). 8. *C. intermedius* (CMN 8704). 9. *C. intermedius* (ROM 776). 10. *C. intermedius* (ROM 777). 11. *C. intermedius* (UALVP 13). 12. *Magnapaulia laticaudus* (LACM 17715). 13. *L. magnicristatus* (CMN 8705). 14. *L. magnicristatus* (TMP 66.4.1). 15. *Parasaurolophus walkeri* (ROM 768). 16. *Olorotitan arharensis* (AEHM 2/845). 17. *L. lambei* (CMN 351). 18. *L. lambei* (CMN 2869). 19. *L. clavinitialis* (CMN 8703). 20. *L. lambei* (FMNH FR380). 21. *L. lambei* (ROM 794). 22. *L. clavinitialis* (TMP 81.37.1). 23. *L. clavinitialis* (YPM 3222). 24. *Lambeosaurus* sp. (CMN 8633). 25. *L. lambei* (ROM 1218). 26. *Hypacrosaurus altispinus* (CMN 8501). 27. *H. altispinus* (AMNH 5278). 28. *H. altispinus* (CMN 8675). 29. *H. altispinus* (ROM 702). 30. *H. stebingeri* (MOR 549).

**Figure 5:**
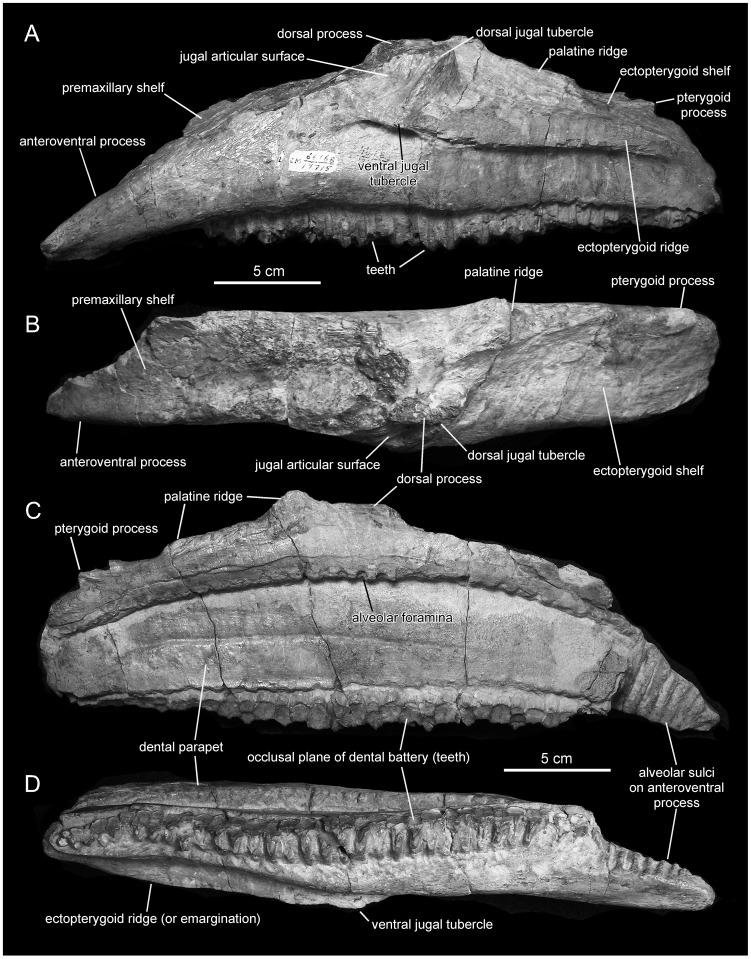
*Magnapaulia laticaudus*, LACM 17715 (holotype), left maxilla. Maxilla in lateral (A), dorsal (B), medial (C), and ventral (D) views.

**Figure 6:**
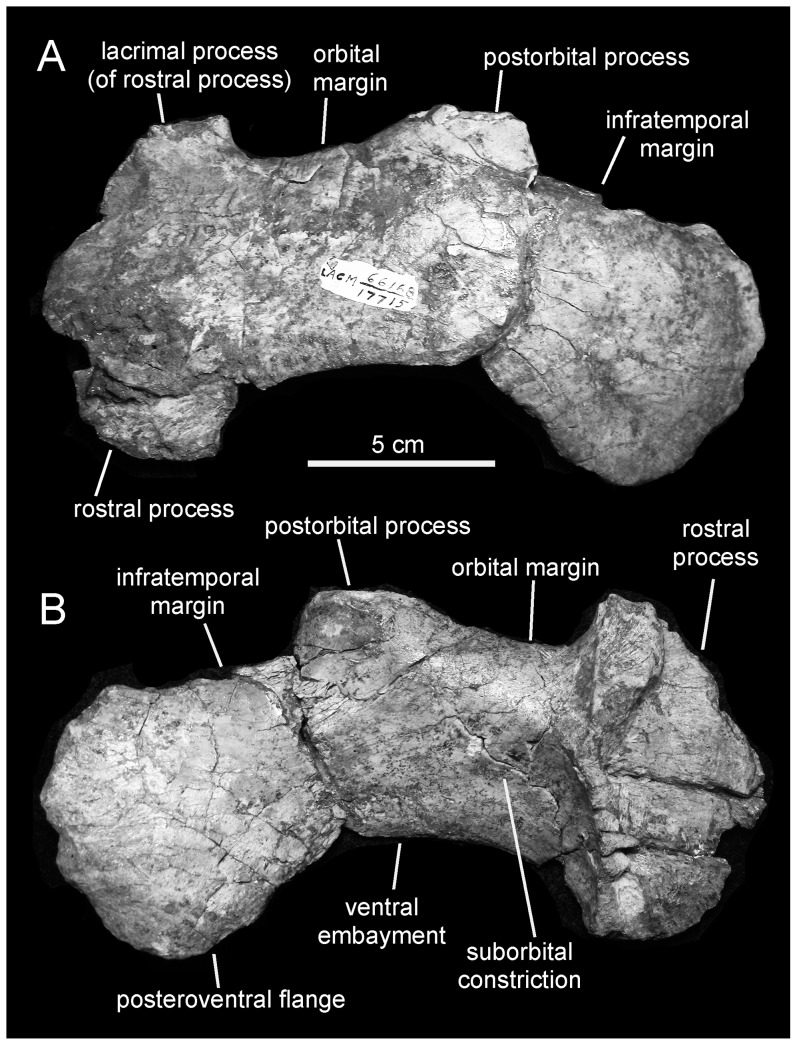
*Magnapaulia laticaudus*, LACM 17715 (holotype), left jugal. Jugal in lateral (A) and medial (B) views.

**Figure 7:**
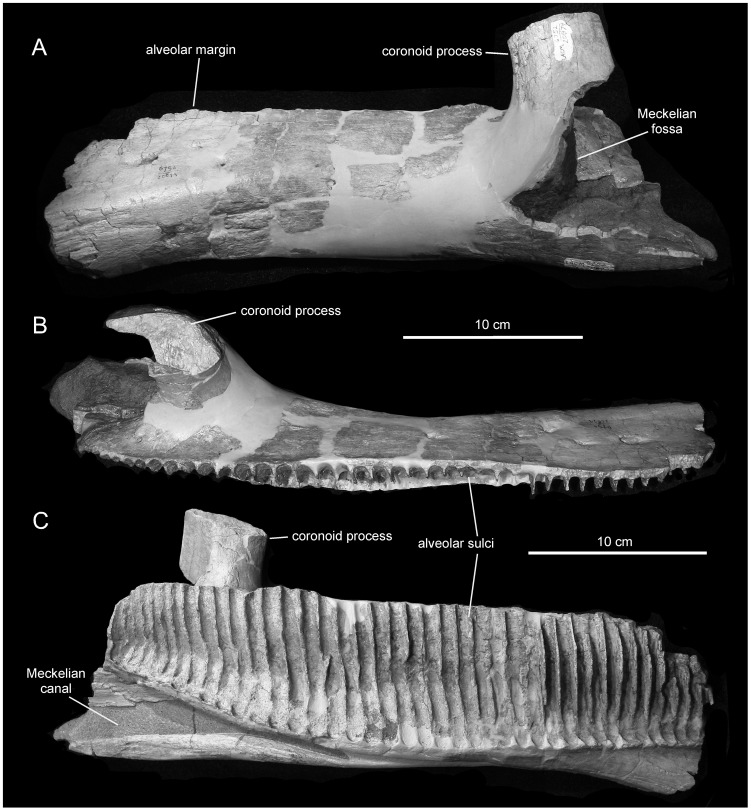
*Magnapaulia laticaudus*, LACM 20874, left dentary. Dentary in lateral (A), dorsal (B), and medial (C) views.

**Figure 8:**
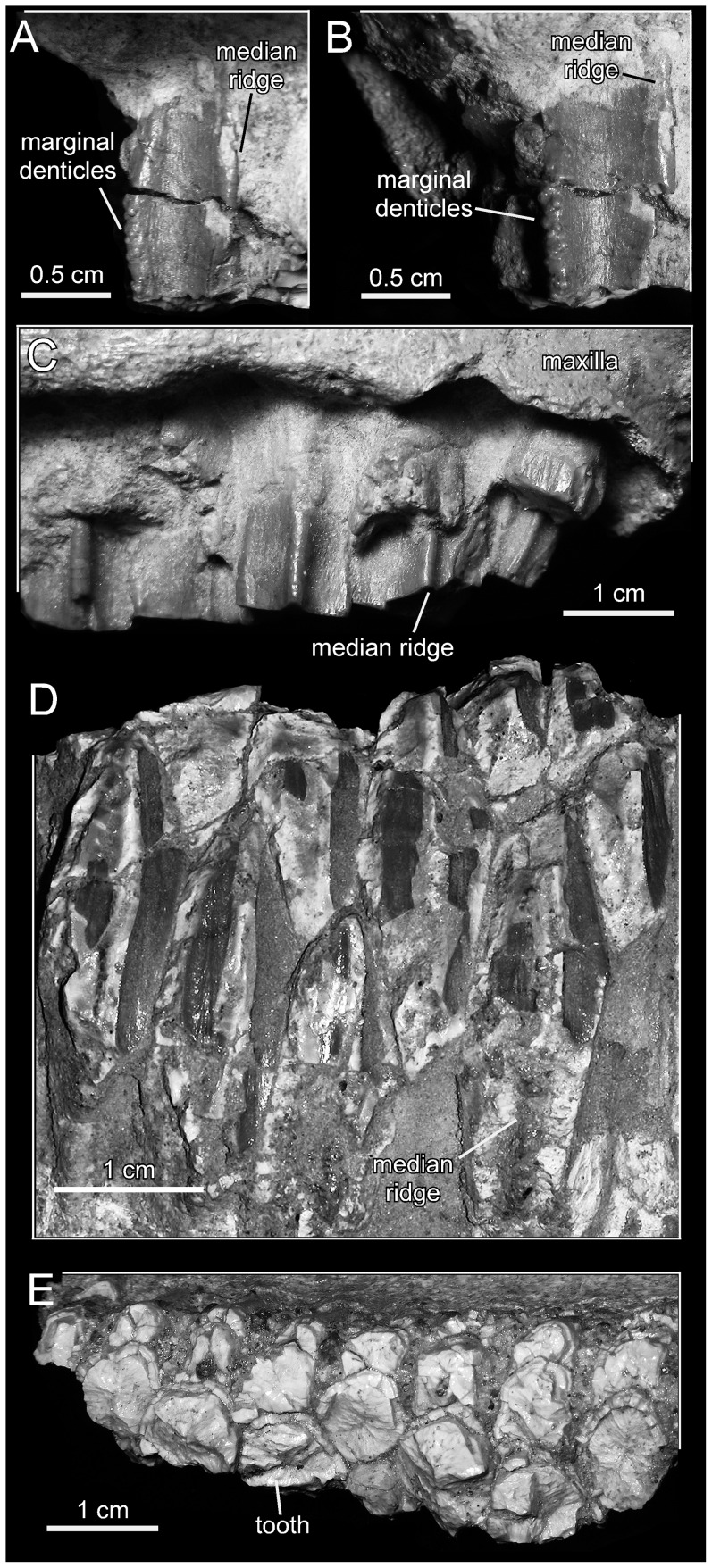
*Magnapaulia laticaudus*, dentition. LACM 17715 (holotype), rostral maxillary tooth crown in labial (A) and mesial (B) views. LACM 17715, caudal maxillary teeth in labial view view (C). LACM 17713, dentary teeth in lingual view (D). LACM 17713, occlusal plane of the dentary dental battery (E).

**Figure 9:**
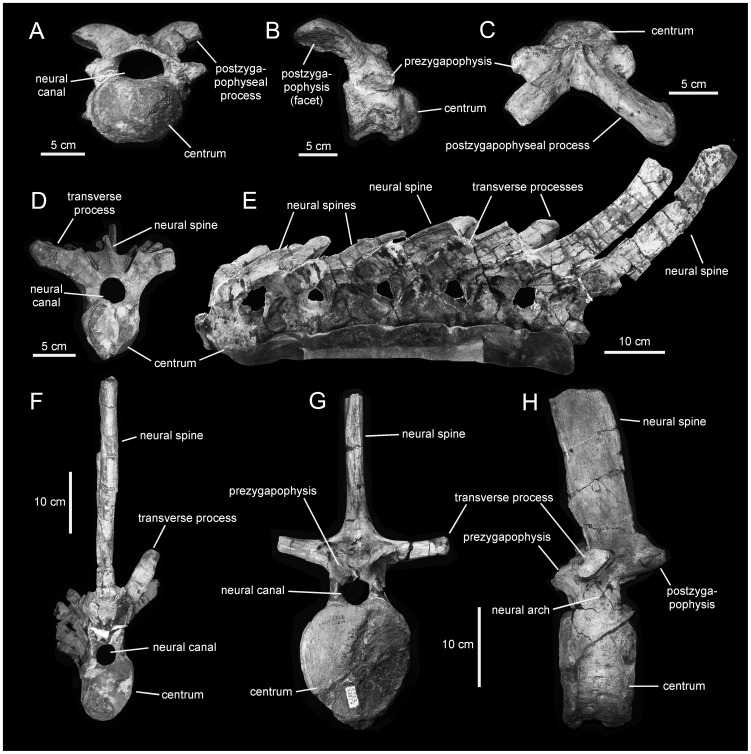
*Magnapaulia laticaudus*, selected pre-sacral vertebrae. A. Cervical vertebra of LACM 17715 (holotype) in cranial view. B. Right lateral view of same. C. Dorsal view of same. D. Cranial dorsal vertebra of LACM 17707 in cranial view. E. Articulated series of cranial dorsal vertebrae, LACM 17707, in left lateral view. F. Cranial dorsal vertebra of LACM 17707 in caudal view. G. Caudal dorsal vertebra of LACM 20874 in cranial view. H. Left lateral view of same.

**Figure 10:**
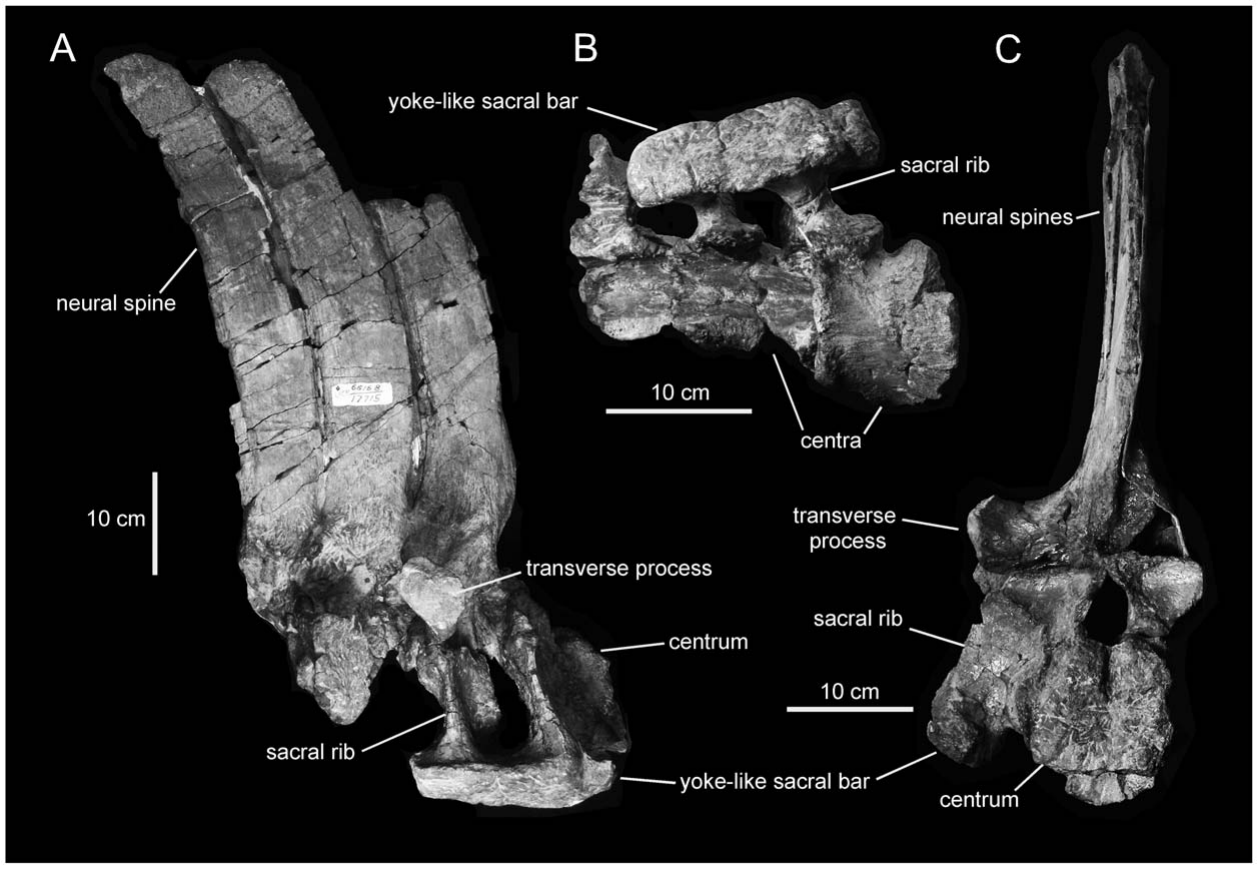
*Magnapaulia laticaudus*, LACM 17715 (holotype), partial sacrum. Sacrum in right lateral (A), ventral (B), and cranial (C) views.

**Figure 11:**
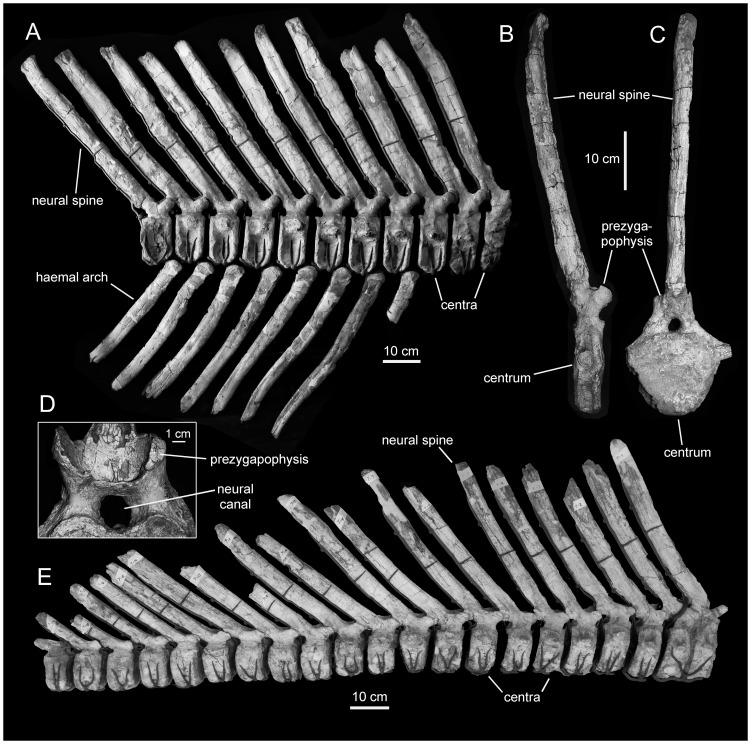
*Magnapaulia laticaudus*, caudal vertebrae. LACM 17705, proximal caudal vertebrae in right lateral view (A). LACM 17702, proximal caudal vertebra in right lateral (B) and cranial (C) views. LACM 20873, proximal to middle articulated caudal vertebrae in right lateral view (D).

**Figure 12:**
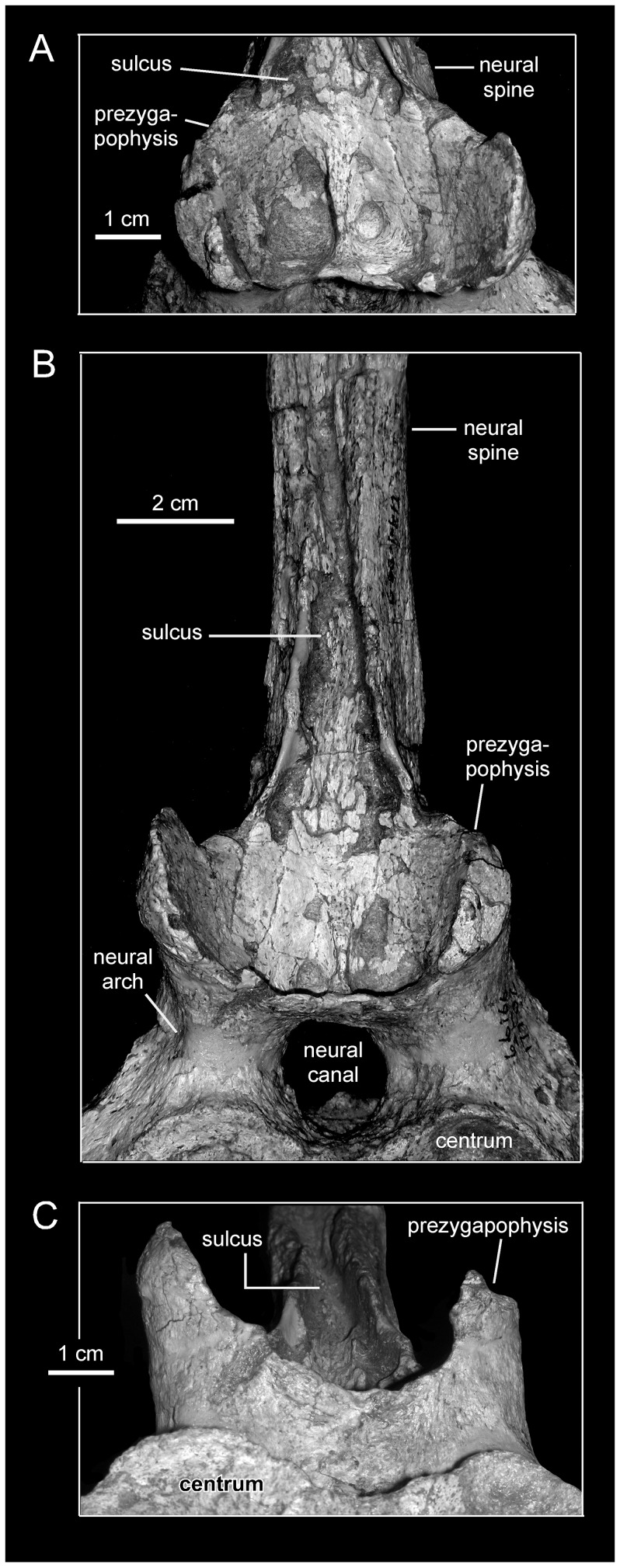
*Magnapaulia laticaudus*, LACM 17702, detail of the prezygapophyses and the base of the neural spine of a proximal caudal vertebra. Vertebra in dorsal (A), cranial (B), and ventral (C) views.

**Figure 13:**
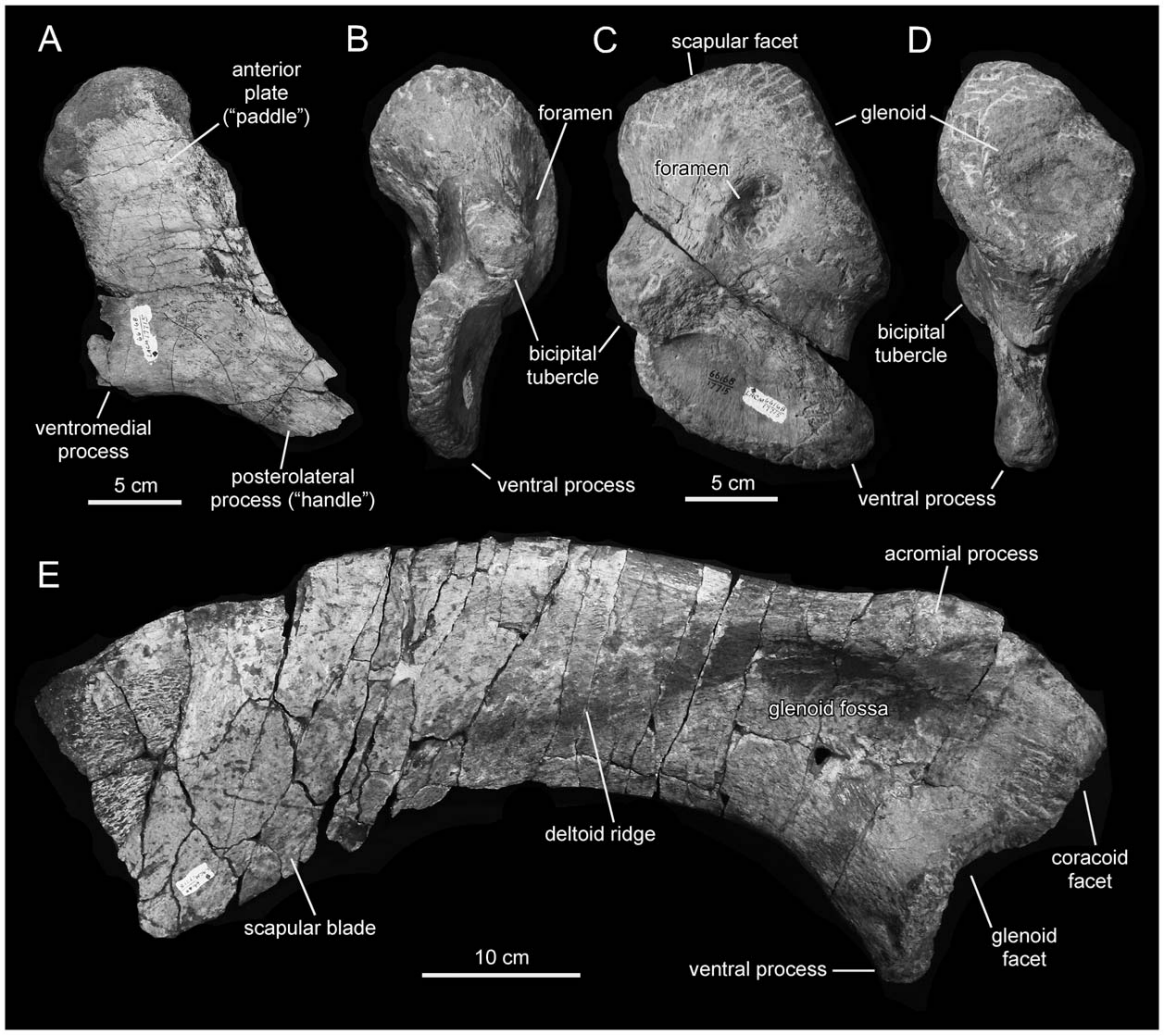
*Magnapaulia laticaudus*, LACM 17715 (holotype), axial elements and pectoral girdle. Left sternum in ventral view (A). Left coracoid in cranial (B), lateral (C), and caudal (D) views. Right scapula in lateral view (E).

**Figure 14:**
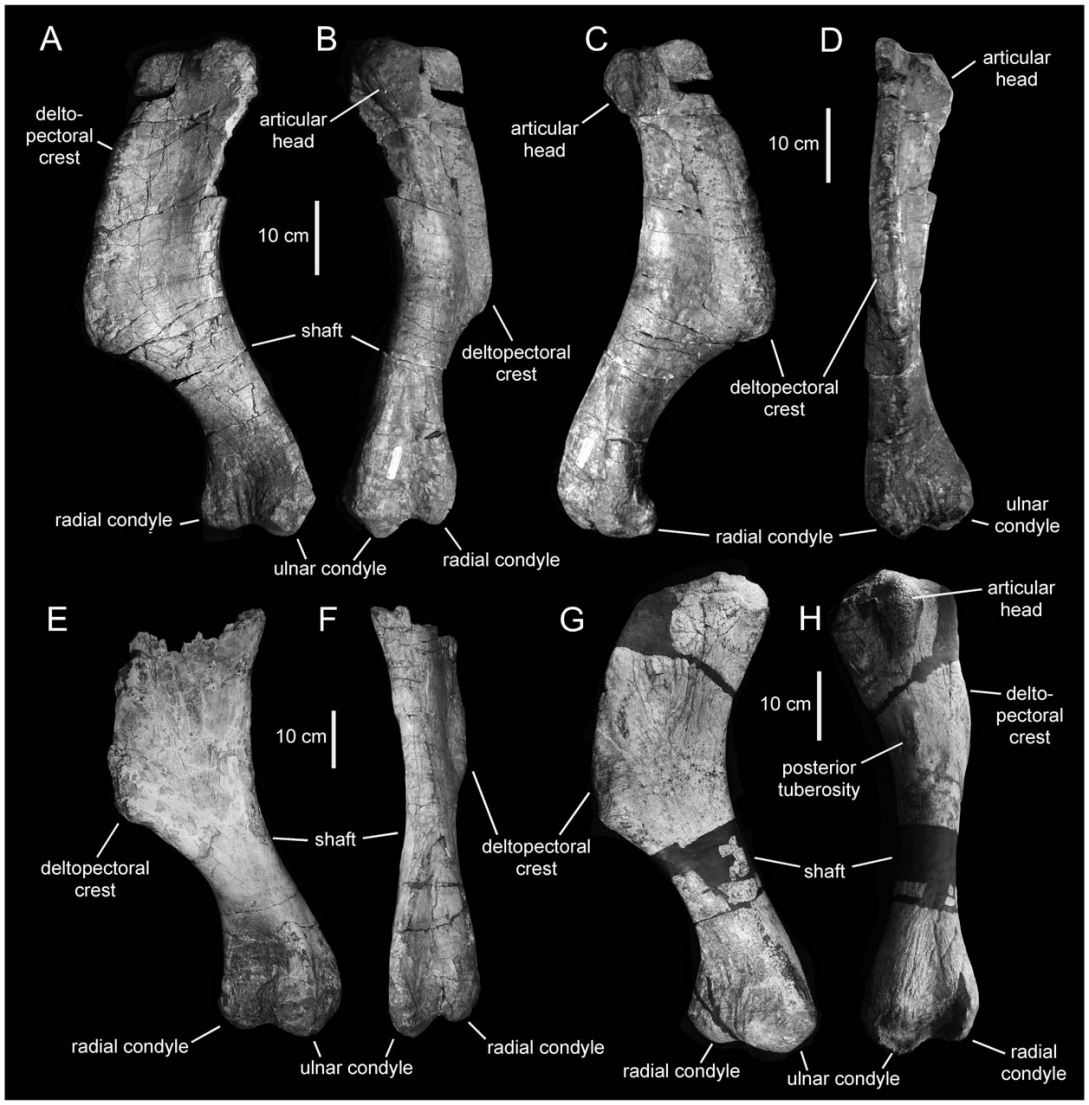
*Magnapaulia laticaudus*, humeri. LACM 17715 (holotype), right humerus in craniomedial (A), caudal (B), caudolateral (C), and cranial (D) views. LACM 17712, partial right humerus in craniomedial (E) and caudal (F) views. LACM 17716, right humerus in craniomedial (G) and caudal (H) views.

**Figure 15:**
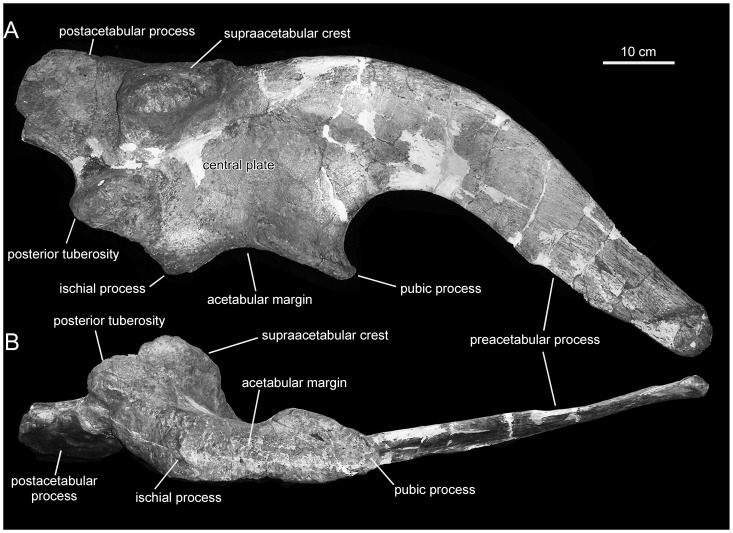
*Magnapaulia laticaudus*, LACM 20874, right ilium. Ilium in lateral (A) and ventral (B) views.

**Figure 16:**
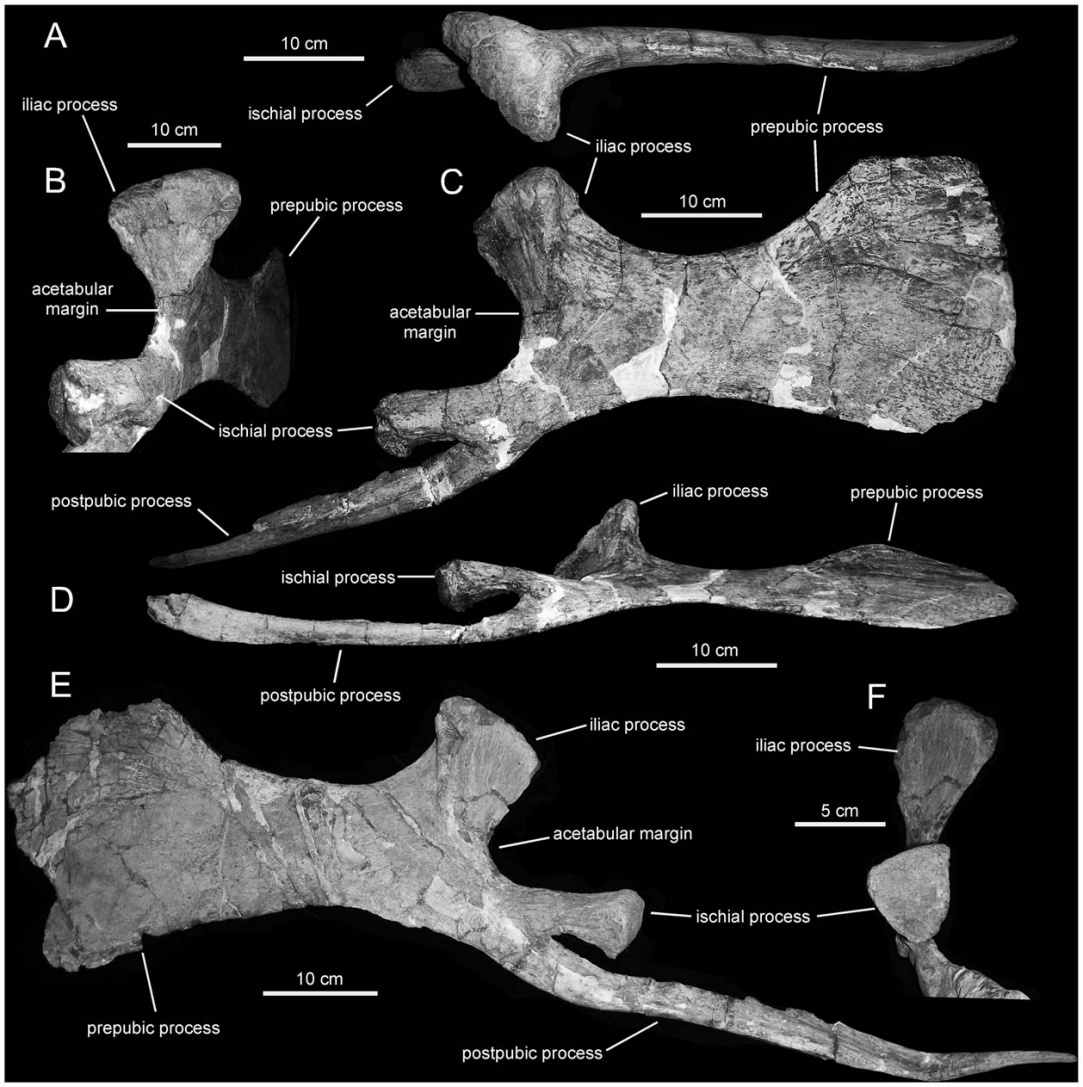
*Magnapaulia laticaudus*, LACM 20874, pubes. Right pubis in dorsal (A), caudolateral (B), lateral (C), and ventral and slightly lateral (D) views. Left pubis in lateral (F) and caudal (H) views.

**Figure 17:**
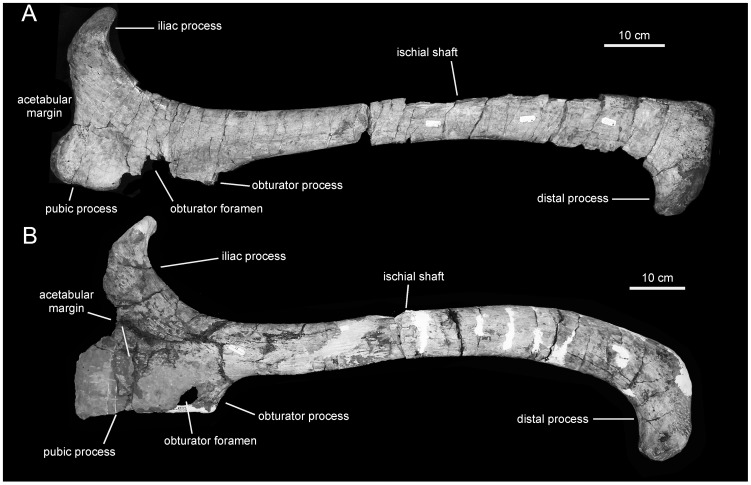
Lambeosaurine ischia from Baja California, Mexico. *Magnapaulia laticaudus* left ischium, LACM 17715 (holotype), in lateral view (A). Left ischium of an indeterminate lambeosaurine dinosaur from La Bocana Roja Formation, LACM 28990, in lateral view (B).

**Figure 18:**
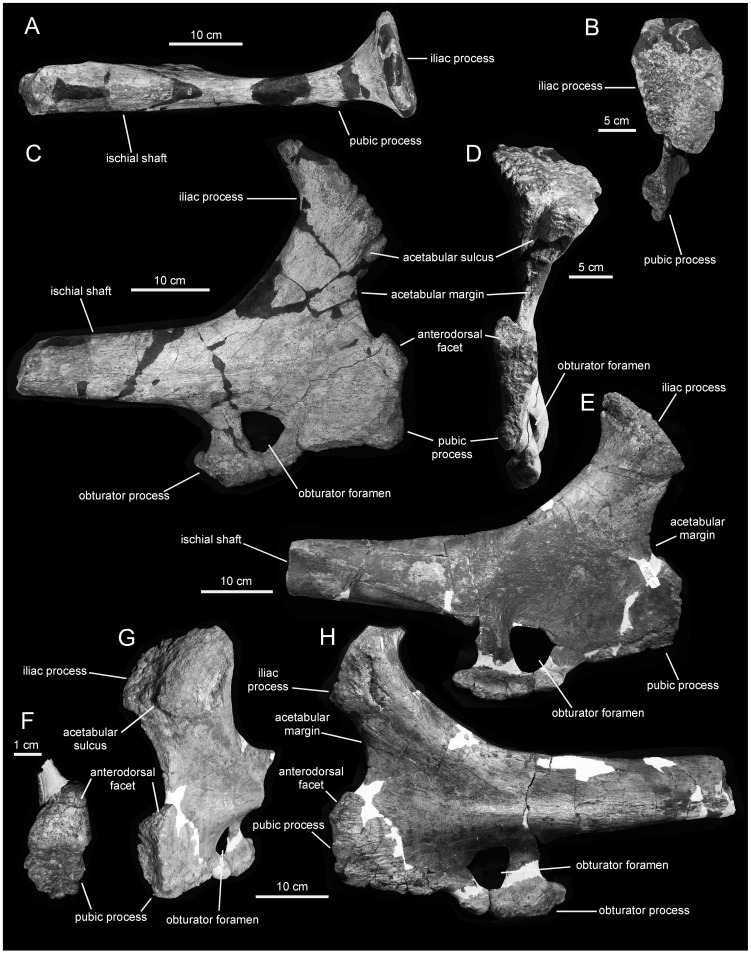
*Magnapaulia laticaudus*, LACM 20874, proximal ischia. Right ischium in dorsal (A), craniodorsal (B), lateral (C), and cranial (D) views. Left ischium in medial (E), craniolateral (G), and lateral (H) views. Pubic process of left ischium showing craniodorsally-facing triangular facet (F).

**Figure 19:**
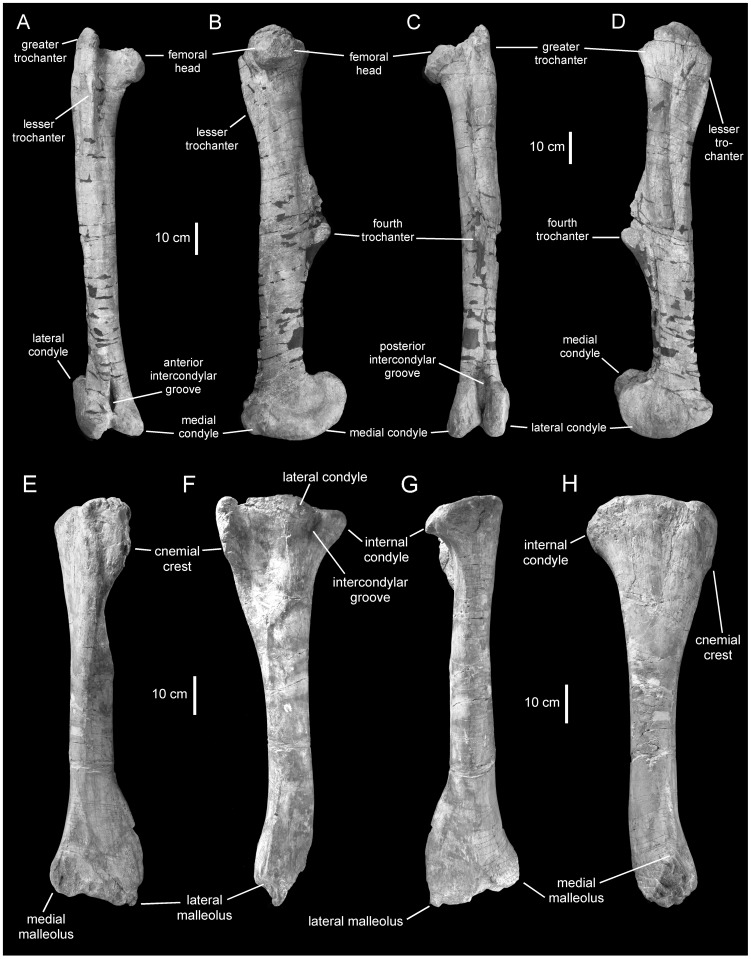
*Magnapaulia laticaudus*, hindlimb elements. LACM 17715 (holotype), right femur in cranial (A), medial (B), caudal (C), and lateral (D) views. LACM 20876, left tibia in cranial (E), lateral (F), caudal (G), and medial (H) views.

**Figure 20:**
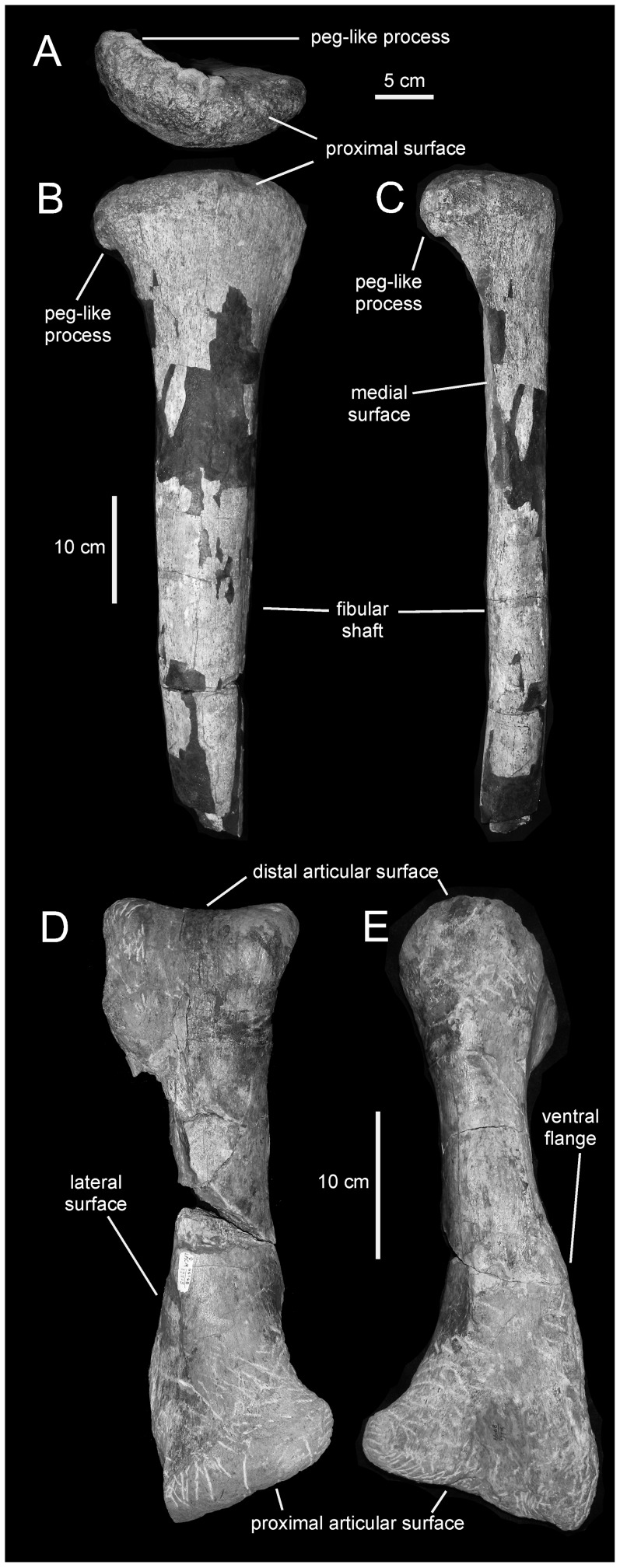
*Magnapaulia laticaudus*, hindlimb elements. LACM 20874, proximal half of the left fibula in proximal (A), lateral (B), and cranial (C) views. LACM 17715 (holotype), right metatarsal III in dorsal (D) and medial (E) views.

**Figure 21:**
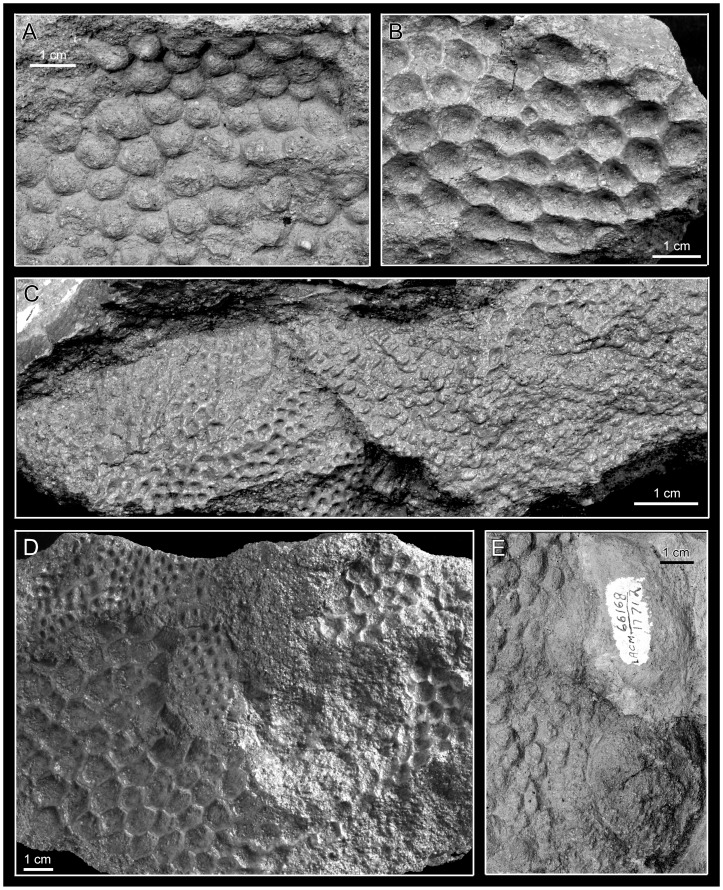
*Magnapaulia laticaudus*, LACM 17712, integumentary structures. Natural cast of scales (A). Scale impressions (B). Casts and impressions of smaller scales (C). Impressions of the co-occurrence of small and large scales in a single skin patch (D). Natural cast of scales adjacent to the cast of a possible osteoderm (arrow) (E).

#### Revised Diagnosis

Lambeosaurine hadrosaurid possessing the following autapomorphies: longest haemal arches of proximal caudal vertebrae being at least four times longer than the depth of their respective centra, and the base of prezygapophyses in caudal vertebrae merging to form a bowl-shaped surface, which, in the proximal-most caudals, is continuous dorsally with a deep sulcus on the cranial surface of the neural spine. In addition, *Magnapaulia laticaudus* is characterized by the following unique combination of characters: downturned rostroventral process of the maxilla (convergent in *Tsintaosaurus spinorhinus*, *Angulomastacator daviesi*, and *Olorotitan arharensis*), the ventral margin of which forms a 18-degree angle with the alveolar margin of the element at its mid-length; tear-shaped external naris with length/width ratio between 1.85 and 2.85, being rostrocaudally longer than in *Hypacrosaurus altispinus* but shorter than in *Lambeosaurus* spp., *Corythosaurus* spp., and *H. stebingeri*, and mediolaterally wider than in *Parasaurolophus walkeri* (condition shared with *Velafrons coahuilensis* and convergent in *Olorotitan arharensis*); and greatly elongated neural spines (at least four times the depth of their respective centra) of dorsal, sacral, and proximal caudal vertebrae (convergent in *Hypacrosaurus* spp. for the dorsal neural spines and *Barsboldia sicinskii* for the sacral and proximal caudal neural spines).

#### Holotype

LACM 17715, consisting of left premaxilla, left maxilla, left jugal, partial atlas, three partial and two nearly complete cranial to middle cervical vertebrae, one nearly complete caudal cervical vertebra, two cervical postzygapophyseal processes, two nearly complete cranial-most dorsal vertebrae, two cranial dorsal neural arches, two partial cranial dorsal vertebrae, three cranial dorsal centra, one fragment of cranial neural arch, two fragments of middle to caudal dorsal vertebrae, two caudal dorsal neural arches, one caudal dorsal centrum, partial sacrum composed of four co-ossified vertebrae (three neural arches with neural spines and one centrum), two pairs of co-ossified sacral centra, one caudal sacral vertebra lacking the centrum, one sacral centrum, one sacral neural spine, six fragmentary sacral ribs, two caudal neural arches, partial right sternum, left coracoid, right scapula, right humerus, left ischium, complete left femur and fragmentary femur, proximal end of left tibia, and a nearly complete left metatarsal III (Fig. 2). Measurements of LACM 17715 are given in Tables 1–3.

**Table 1:** Selected cranial measurements (in mm) of *Magnapaulia laticaudus*.

Element	Measurement
Premaxilla (LACM 17715), length along long axis of external naris	353
Premaxilla (LACM 17715), maximum width of oral margin	132
Premaxilla (LACM 17715), length of external naris	260
Maxilla (LACM 17715), length	301
Maxilla (LACM 17715), maximum height as preserved, perpendicular to and excluding the tooth row	78
Jugal (LACM 17715), rostrocaudal length	178
Jugal (LACM 17715), minimum depth of orbital constriction	53
Jugal (LACM 17715), depth from lacrimal process to ventral apex of rostral process	84
Dentary (LACM 20874), length of preserved dental battery	323
Dentary (LACM 20874), depth of lateral dentary ramus at midlength of dental battery	86

**Table 2:** Selected appendicular measurements (in mm) of *Magnapaulia laticaudus*.

Element	Measurement
Coracoid (LACM 17715), length from scapular facet to apex of ventral process	213
Coracoid (LACM 17715), width from glenoid to biceps tubercle	142
Coracoid (LACM 17715), glenoid facet, mediolateral width	89
Scapula (LACM 17715), length from dorsal margin of coracoid facet to dorsodistal corner of partial scapular blade	611
Scapula (LACM 17715), width from dorsal margin of pseudoacromion process to apex of glenoid facet	216
Humerus (LACM 17715), length from articular head to ulnar condyle	598
Humerus (LACM 17715), width perpendicular to maximum expansion of deltopectoral crest	160
Humerus (LACM 17716), length from articular head to ulnar condyle	650
Humerus (LACM 17716), width perpendicular to maximum expansion of deltopectoral crest	173
Humerus (LACM 17712), length from incomplete proximal region to ulnar condyle	723 (803)
Humerus (LACM 17712), width perpendicular to maximum expansion of deltopectoral crest	242
Ilium (LACM 20874), length from distal end of preacetabular process to incomplete proximal region of postacetabular process (measured parallel to plane containing the ventral margins of the ischiac and pubic peduncles)	912
Ilium (LACM 20874), height from maximum convexity of dorsal iliac margin to apex of pubic peduncle	291
Ilium (LACM 20874), length of iliac plate, from anteroventral tip of pubic peduncle to posterior tuberosity of ischiac peduncle (measured parallel to plane containing the ventral margins of the ischiac and pubic peduncles)	367
Ischium (LACM 17715), length from pubic peduncle to distal process	1153
Ischium (LACM 17715), height from iliac peduncle to ventral margin of pubic peduncle	318
Ischium (LACM 17715), height of ventral projection of distal process	203
Ischium (LACM 20874), height from iliac peduncle to ventral margin of pubic peduncle	414
Pubis (LACM 20874), length from maximum concavity of acetabular margin to cranial margin of prepubic blade	423
Pubis (LACM 20874), length from cranial margin of prepubic blade to distal end of postpubic process	728
Pubis (LACM 20874), maximum height of prepubic blade	233
Pubis (LACM 20874),height from iliac peduncle to ventral margin of proximal region of ischiac peduncle	237
Pubis (LACM 20874), height of prepubic constriction perpendicular to maximum concavity of dorsal margin	119
Femur (LACM 17715), length	1232
Femur (LACM 17715), width of lateral condyle	270
Femur (LACM 17715), craniocaudal diameter of femoral shaft proximal to fourth trochanter	141
Tibia (LACM 17706), length	1259
Tibia (LACM 17706), width of proximal end	406
Tibia (LACM 17706), craniocaudal midshaft diameter	144
Tibia (LACM 17711), length	1245
Tibia (LACM 17711), intercondylar width of distal end	278
Tibia (LACM 17711), craniocaudal midshaft diameter	123
Tibia (LACM 20876), width of proximal end	374
Tibia (LACM 20874), width of proximal end	270
Fibula (LACM 29303), length	993
Fibula (LACM 29303), width of partially preserved proximal end	151
Fibula (LACM 29303), craniocaudal midshaft diameter	68
Fibula (LACM 17717), length	967
Fibula (LACM 17717), craniocaudal midshaft diameter	59
Fibula (LACM 20874), width of proximal end	205
Metatarsal III (LACM 17715), length	450
Metatarsal III (LACM 17715), height of proximal surface	168
Metatarsal III (LACM 17715), width of distal surface	129

The number between parentheses was estimated using Fig. 24.

**Table 3:** Selected vertebral measurements (in mm) of *Magnapaulia laticaudus*.

Element	Measurement
Sacrum (LACM 17715), centrum height of most complete, anterior-most one of partially preserved series	122
Sacrum (LACM 17715), neural spine height of most complete, posterior-most of partially preserved series	483
Sacrum (LACM 17715), centrum craniocaudal width of most complete, anterior-most one of partially preserved series	87
Proximal caudal vertebrae (LACM 17702), centrum height	148
Proximal caudal vertebrae (LACM 17702), neural spine height	545
Proximal caudal vertebrae (LACM 17702), centrum craniocaudal width	55
Proximal to middle caudal vertebrae (LACM 20873; eighth vertebra of preserved series), centrum height	124
Proximal to middle caudal vertebrae (LACM 20873; eighth vertebra of preserved series), neural spine height	476
Proximal to middle caudal vertebrae (LACM 20873; eighth vertebra of preserved series), centrum craniocaudal width	72
Proximal caudal vertebrae (LACM 17705), centrum height	151
Proximal caudal vertebrae (LACM 17705), neural spine height	580
Proximal caudal vertebrae (LACM 17705), centrum craniocaudal width	75
Proximal caudal vertebrae (LACM 17705), haemal arch length	562

#### Referred specimens

LACM 17698 (cervical vertebra), 17699 (tooth), 17700 (tooth), 17702 (cervical and dorsal rib fragments, one proximal and one distal caudal vertebrae, centrum and neural arch of a proximal caudal vertebra, and eight caudal neural spine fragments), 17703 (left ischium), 17704 (tibia and femoral fragment), 17705 (articulated series of 11 proximal caudal vertebrae), 17706 (left tibia), 17707 (partial right humerus), 17708 (ischium), 17709 (dorsal vertebra), 17710 (proximal region of dorsal rib), 17711 (left tibia), 17712 (dorsal vertebra and numerous integumentary impressions), 17713 (dentary fragment), 17716 (right humerus and humeral fragment), 17717 (partial left fibula), 20873 (articulated series of 21 proximal to middle caudal vertebrae), 20874 (partial left dentary, nearly complete dorsal vertebra, three caudal dorsal centra, twelve fragments of dorsal neural spines, one caudal centrum, nearly complete right ilium, proximal region and shaft fragment of left ischium, left pubis, distal processes of left and right ischia), 20875 (distal processes of left and right ischia), 20876 (left tibia), 20883 (numerous associated vertebral fragments), 20884 (dentary and pubic fragments), and 20885 (partial left fibula). Measurements of selected specimens are given in Tables 1–3. The referral to *Magnapaulia laticaudus* of the above specimens is based on 1) presence of lambeosaurine characters (see description below) in the appendicular elements (e.g., humeri, ischia, pubes) that are also found in the type specimen, in combination with 2) the specimens were found associated with the type specimen within locality LACM 7253. These two reasons together suggest that the most parsimonious interpretation is that these lambeosaurine remains belong to the same taxon. The alternative choice would be to name two distinct taxa from the same locality, because the type LACM 17715 is diagnostic on its own, and so is LACM 17705. However, we think that the first choice of considering a single taxon is the most conservative, simpler explanation given the anatomical data at hand.

#### Occurrence

The type and referred specimens of *Magnapaulia laticaudus* were found in late Campanian strata of the El Gallo Formation, near the western coastline of the state of Baja California Norte, northwestern Mexico (Fig. 1), at LACM locality 7253. This locality lies no more than three miles west of the town of El Rosario (Fig. 1), in the west rim of a mesa north of Arroyo del Rosario. According to LACM archival records, locality 7253 incorporates former locality numbers 66166–66168 and 6751–6756 (see Supporting Information S1). The El Gallo Formation lies above the La Bocana Roja Formation and below the Rosario Formation; the El Gallo Formation is separated from those other two formations by angular unconformities [22]. The lambeosaurine-bearing strata at locality LACM 7253 pertain to the El Disecado Member [7], which has an estimated age of 73.6–73.0 million years [21], [25]. These fossiliferous strata consist of alternating horizons of massive light-brown sandstones and grey siltstones [22], [23]. Although some sandstone bodies have been interpreted as representing beach deposits, most of them appear to have resulted from high energy stream flows occurring during flood episodes, within the greater context of a narrow flood plain crossed by westernward fluvial systems [23]. More specifically, and according to LACM archives, the sedimentary deposits at locality LACM 7253 consist of yellowish brown to gray cross-bedded sandstones with occasional lignite lenses. Stratigraphically, the locality lies above the first tuff outcropping along the north side of Arroyo del Rosario, below the Rosario Formation.

### Description and comparisons

#### Premaxilla

**Figure 22:**
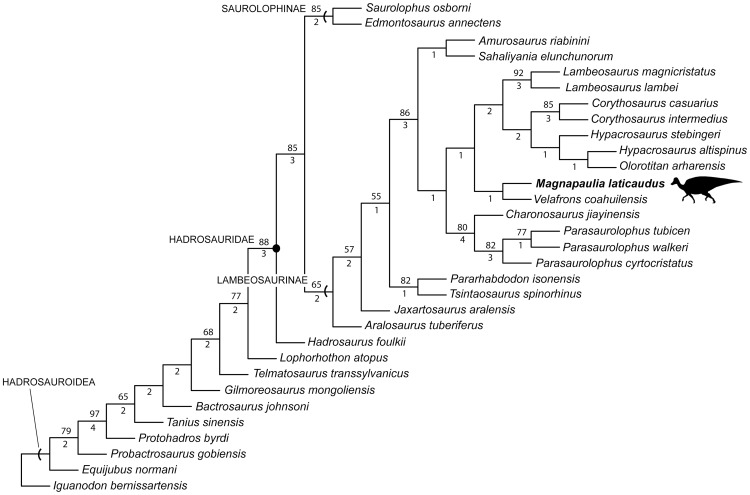
Single most parsimonious tree resulting from maximum parsimony analysis, showing the phylogenetic position of *Magnapaulia laticaudus* within Lambeosaurinae. The numbers above the branches represent bootstrap frequencies, whereas those below are decay indices (Bremer support).

The premaxilla is rostrocaudally elongate and forms the dorsal region of the snout (Fig. 3). Most of the premaxilla is completely preserved, except for the rostromedial border and post-narial regions of the caudal medial and lateral processes. The rostral oral region of the premaxilla is dorsoventrally compressed, whereas the elongated segment composed of the two caudal processes is mediolaterally compressed. The premaxilla of *Magnapaulia laticaudus* shows the general characteristic morphology seen in all other known lambeosaurine premaxillae (except in *Tsintaosaurus spinorhinus*). In particular, the external naris is exposed laterally as an elongated tear-shaped depression entirely confined between the dorsomedial and ventrolateral processes (Fig. 3B). Rostrally, the external naris greatly widens mediolaterally while becoming gradually shallower and confluent rostromedially with the oral margin of the premaxilla. Its length/width ratio is 2.4. Its dorsal surface is smooth and featureless, lacking any trace of foramina or additional excavations. The geometry in laterodorsal view of the external naris differs from that present in *Corythosaurus* spp., *Lambeosaurus* spp., *Hypacrosaurus* spp., *Parasaurolophus walkeri*, and *Olorotitan arharensis* (Fig. 4). The results of the Square-Root Elastic morphometric analysis [33], [34] quantifying the dissimilarity of lambeosaurine external nares show that *M. laticaudus* occupies a different region in the morphospace in relation to that of the other sampled taxa (Fig. 4C). Specifically, the external naris of *M. laticaudus* has a length/width ratio of 2.4, being rostrocaudally more elongate than in *Hypacrosaurus stebingeri* and species of *Corythosaurus* and *Lambeosaurus*. This supports removal of the Baja California lambeosaurine from the genus *Lambeosaurus*. Furthermore, the external naris in *M. laticaudus* is substantially longer than that of *H. altispinus* and it is broader than that of *P. walkeri* (Fig. 4C). The length/width proportions of the external naris of *M. laticaudus* are similar to those of *Velafrons coahuilensis* and *Olorotitan arharensis* [35]: fig. 22 and table D.2).

As is typically seen in hadrosaurids [5], most of the oral region of the premaxilla consists of two layers separated by a narrow cleft (Fig. 3A and 3C). The dorsal layer is rostrally offset relative to the ventral one. Each of the two layers contains four large and triangular denticles per premaxilla. The denticles in the dorsal oral layer are aligned with those of the ventral layer. The dorsal surface of the dorsal layer is rostrocaudally convex and its denticles are ventrally deflected. Only the second most medial denticle is nearly completely preserved in the dorsal layer, whereas the other three are heavily abraded. In the ventral layer, all four denticles are abraded but still recognizable, forming, as preserved, broad and shallow crenulations. Laterally, the two layers merge into a thin rostrolateral margin as the oral region of the premaxilla becomes gradually shallower.

As preserved, the rostrolateral corner of the premaxilla is arcuate and ventrally deflected; however, the lateroventral margin of this area is abraded and therefore might have been more angular than its current gentle profile indicates. The laterodorsal surface of the rostrodorsal corner of the premaxilla is flat and mediolaterally broad (accounting approximately for 25% of the maximum mediolateral breadth of the premaxilla). The laterodorsal surface of the rostrolateral corner of the premaxilla is limited by a sharp rim that bounds laterally the rostral region of the external naris. As the rostrolateral corner of the premaxilla narrows mediolaterally and merges with the rostral oral border, that sharp edge gradually disappears rostrally and the external naris becomes continuous with the rostromedial portion of the oral margin.

Caudally, the rostrolateral corner of the premaxilla is continuous with the long lateral process (Fig. 3B). This process gradually widens mediolaterally as it extends caudodorsally. Its ventral margin describes a gentle concave profile, whereas its dorsal edge is slightly convex in lateral and laterodorsal views. Caudodorsally, the lateral process converges with the medial process of the premaxilla. The medial process also becomes mediolaterally wider as it extends caudodorsally; however, its dorsal and ventral margins are relatively straight when compared to the more curved dorsal and ventral margins of the lateral process. Both processes reach equal mediolateral widths at their caudal-most preserved ends. The inner margins of the two processes, which delimit the external naris, are sharp and well defined. The cross sections of both processes are elliptical.

#### Maxilla

The maxilla is rostrocaudally elongate and shows a subtriangular lateral profile (Fig. 5A). The element is nearly complete, missing only most of the dorsal process and part of the dorsal margin of the rostrodorsal surface, as well as the majority of the mediodorsal border of the palatine process and the distal end of the pterygoid process.

As preserved, the rostral third of the maxilla is relatively shallow and triangular in lateral view, so that the rostrodorsal region of the maxilla forms a 25-degree angle with the ventral alveolar margin along that same region of the bone. However, the area adjacent and rostral to the dorsal process is abraded, which likely contributes to the apparent shallow profile of the rostrodorsal region of the maxilla. As in all lambeosaurines, there is no rostromedial process medially offset from the rostral body of the maxilla, but instead a ventrally slopping shelf that would support and underlie the caudoventral process of the premaxilla (Fig. 5B). This premaxillary shelf is mediolaterally extensive and faces dorsally and slightly laterally, so that its surface is obliquely exposed in lateral view. Along its caudal half, the dorsal articular surface of the premaxillary shelf is gently concave mediolaterally. This dorsal relief gradually becomes flat along the rostral half of the shelf. The medial margin of the shelf forms a prominent flange above the arcuate row of alveolar foramina that borders the dental parapet on the medial surface of the maxilla. However, this flange is abraded, particularly along its rostral half, where it appears largely incomplete. The dentigerous rostral fourth (rostroventral process) of the maxilla of *Magnapaulia laticaudus* is remarkable in being ventrally deflected (Fig. 5A) more so than in other lambeosaurines, with exception of *Tsintaosaurus spinorhinus* (e.g., IVPP V725), *Angulomastacator daviesi* [9], and *Olorotitan arharensis* [36], [37]. This deflection is such that the ventral alveolar margin of the rostral fourth of the maxilla forms an 18-degree angle with the alveolar margin at mid-length of the maxilla, producing a characteristic pendant profile of the rostral tip of the bone. Outside Hadrosauridae, a downturned rostroventral maxillary process is found in *Shuangmiaosaurus gilmorei* [38].

The base of the dorsal process is approximately centered on the mid-length of the maxilla. Only the caudoventral extent of the articular surface for the jugal is preserved, whereas that for the lacrimal is missing. However, enough is preserved to indicate that the jugal articular surface is as in all other hadrosaurids (except *Pararhabdodon* and *Tsintaosaurus*; see [39]): a subtriangular facet that is bounded caudally by a thick and prominent border, which terminates ventrally into a laterally protruding jugal tubercle (Fig. 5A). This ventral jugal tubercle is continuous with the rostral end of the lateral ridge of the ectopterygoid shelf. The caudal border of the jugal articular surface is rostroventrally (obliquely) oriented, bounding a shallow embayment that exists between this and the ectopterygoid shelf. The preserved portion of the jugal articular surface is triangular and depressed throughout its caudodorsal region. This surface faces dorsolaterally and contains dorsoventrally and slightly obliquely oriented thin and sharply defined ridges. Ventrally, the jugal articular surface is bounded by a thick and longitudinal ridge that is continuous caudally with the ectopterygoid emargination. Below that longitudinal ridge, the lateral surface of the maxilla is pierced by two large foramina. These foramina are approximately equal in size and rostrocaudally elongate. Another much smaller foramen is present further rostrally and dorsally. No other foramina are apparent, aside from the large rostral foramen present in all hadrosauroids; that in *Magnapaulia laticaudus*, as in other lambeosaurines, opens on the dorsal surface just medial to the articular surface for the lacrimal and jugal, along the premaxilla-maxilla contact, and is not exposed laterally when the maxilla is articulated in the skull.

The ectopterygoid shelf accounts for 45% of the total length of the maxilla (measured along the entire alveolar margin) (Fig. 5A). The lateral margin of the shelf is parallel to the caudal extent of the alveolar margin, as is typically observed in Lambeosaurinae and most other hadrosaurids except *Brachylophosaurus canadensis*, *Maiasaura peeblesorum*, and *Wulagasaurus dongi* [40], [41]. The dorsal surface of the ectopterygoid shelf is slightly concave mediolaterally (Fig. 5B). It gradually changes orientation from being dorsally and slightly laterally facing along its rostral region to face dorsally at the caudal end of the maxilla. In addition, the shelf progressively becomes mediolaterally narrower caudally, so that it is 1.85 wider rostrally than at the caudal end of the maxilla. The lateral ridge of the ectopterygoid shelf is dorsoventrally thick and very prominent, as in all hadrosaurids. Its ventral border is sharply defined, whereas its dorsal margin is only well defined along the caudal third of the ridge; in contrast, its rostral half is smooth and merges with the dorsal surface of the ectopterygoid shelf. The lateral surface of the caudal region of the maxilla is deeply inset medially below and relative to the ectopterygoid ridge. At the opposite side of the ectopterygoid emargination is the palatine ridge to which the homonymous bone attaches (Fig. 5C). This is a prominent (although abraded in LACM 17715) and mediodorsally expanded ridge that bounds the ectopterygoid shelf medially. At the caudomedial end of the maxilla there is the finger-shaped and mediolaterally compressed pterygoid process (Fig. 5B). Only the proximoventral region of this structure has been preserved in LACM 17715.

The medial surface of the maxilla is flat and consists mostly of the dental parapet (Fig. 5C). A longitudinal and shallow vascular groove bisects the dental parapet in two nearly equally dorsal and ventral halves along the caudal half of the maxilla. The dental parapet is dorsally limited by a gently arcuate row of alveolar foramina. This row approximately divides the medial side of the maxilla into a dorsal third and the ventral two thirds. Postdepositional compression has pushed the parapet region of the medial side of the maxilla laterally, so that the dorsal third above the row of alveolar foramina appears medially offset.

There are 41 tooth positions, of which only the seven most rostral alveoli did not preserve teeth (Fig. 5D). The alveoli nearly extend to the rostral tip of the maxilla.

#### Jugal

The only known jugal is missing the rostral-most margin of the rostral process, parts of the dorsal (lacrimal process) and ventral edges of the rostral process, nearly the entire postorbital process, the dorsal margin of the caudal constriction, and the quadratojugal process (Fig. 6). As preserved, the rostral process is approximately twice as deep as it is rostrocaudally broad and D-shaped, as in all known lambeosaurines except *Tsintaosaurus spinorhinus* (e.g., IVPP V830), *Hypacrosaurus altispinus* (e.g., ROM 702) and *Parasaurolophus* spp. (e.g., ROM 768, NMMNH P-25100). Although incomplete, the lacrimal process and the ventral corner of the rostral process are much expanded dorsally and ventrally. The medial articular surface of the rostral process is medially recessed and bounded caudally by a very prominent and thick vertical ridge (Fig. 6B). Along its dorsal third, this ridge widens rostrocaudally to form an arcuate facet.

Rostrally, the jugal of *Magnapaulia laticaudus* shows a proportionately deep orbital constriction, comparable in depth to that of *Amurosaurus riabinini* [42]. The dorsal and ventral margins of the orbital constriction in *M. laticaudus* are parallel to each other and nearly straight rostral to the base of the postorbital process and the rostroventral edge of the jugal flange, respectively (Fig. 6A). The base of the postorbital process is rostrocaudally wide, accounting for slightly more than 20% of the total length of the jugal. The infratemporal and ventral margins of the caudal constriction are slightly incomplete; as preserved, the orbital and infratemporal (caudal) constrictions are approximately equal in depth. The lateral profile of the ventral embayment of the jugal, which extends between the rostral process and the caudoventral flange, is very pronounced, as in other lambeosaurines [41]. Most of the caudoventral flange is preserved, with exception of its most ventral edge and the caudal margin that would be continuous with the quadratojugal flange. The caudoventral flange appears to have been only moderately expanded; as preserved, it is 1.25 the depth of the infratemporal constriction.

#### Dentary

The dentary is represented by a partial element missing the caudal and rostral-most extents of the dental battery along with the rostral edentulous region, all teeth, the caudolateral wall below the coronoid process, and the dorsal expansion of the latter process (Fig. 7). The dentary of *Magnapaulia laticaudus* is relatively elongate: the ratio between the total length of the element (from the level of the caudal end of the coronoid process to the most rostral preserved margin) and its dorsoventral depth at mid-length of the dental battery is over 3.5. The preserved edentulous portion the dentary is not deflected more than 15 degrees relative to the ventral margin of the rostral series of alveoli (Fig. 7A). It is, however, possible that when complete the rostral region of this dentary reached a greater degree of deflection. The ventral deflection of the dentary is estimated to originate between two thirds and 70% of the length of the dental battery (length measured taking the caudal margin of the coronoid process as starting point). Although it is somewhat variable in hadrosaurids [41], the ventral deflection in various dentaries of *Corythosaurus* spp. (e.g., ROM 776, 871), *Hypacrosaurus altispinus* (e.g., ROM 702), *Lambeosaurus lambei* (e.g., CMN 8703), or *Charonosaurus jiayinensis* (uncatalogued CUST specimen) originates at a similar level than in *M. laticaudus*.

On the lingual side of the dentary the dental parapet is missing, exposing a total of 39 alveoli (Fig. 7C). However, the total count would probably exceed 40 tooth positions when considering the unpreserved rostral region of the dentary. Each alveolar sulcus is bounded by sharp and thin laminae. The alveoli become slightly narrower toward the rostral and caudal ends of the dental battery. Along the caudal two thirds of the dental battery, the long axes of the alveoli are slightly tilted caudally. The caudal opening of the Meckelian canal is very broad; however, the canal abruptly narrows rostrally, at approximately 12 tooth positions from the caudal end of the dental battery.

The lateral surface of the dentary is dorsoventrally convex. This convexity becomes particularly accentuated along the rostral third of the dentary with the formation of a longitudinal and smooth ridge, which is parallel to the ventral margin of the bone (Fig. 7A). Caudal to the rostral deflection, the ventral margin of the dentary is straight, with the exception of the area just rostral to the base of the coronoid process; this area is gently bowed, as is commonly observed in other lambeosaurines and some saurololophine hadrosaurids [41]. Although incomplete, enough is preserved of the coronoid process to show that, as typically occurs in hadrosaurids [41], its long axis is rostrally inclined. In addition, the dentary of *Magnapaulia laticaudus* shows several other characters that are always present in hadrosaurids, such as a substantial lateral offset of the coronoid process from the dental battery, the caudal extension of the dental battery beyond the caudal margin of the coronoid process, and an rostrocaudally straight dorsal alveolar margin that is parallel to the lateral wall of the dentary (Fig. 7B).

#### Dentition

Dentary teeth are known from an eroded fragment of dental battery (LACM 17713). The dentary occlusal plane shows a maximum of three teeth arranged mediolaterally (Fig. 8E). There are at least three teeth per alveolus arranged dorsoventrally in the dental battery. Tooth crowns are lanceolate, with a height/width ratio of 3.2 (Fig. 8D). The enameled lingual surface of some of the best-preserved teeth bears a primary median carina and at least one fainter secondary ridge. The margins of these tooth crowns are too incompletely preserved to observe the shape and size of denticles.

Several maxillary teeth are preserved in situ in the maxilla of LACM 17715; thus, only part of their enameled labial sides is exposed (Fig. 8A–8C). The occlusal plane is composed of two teeth arranged mediolaterally from at least the thirteenth to the thirty-fourth position. Only a single median carina is observed in these teeth. Marginal denticles are curved and mammilated structures (Fig. 8A). Each denticle is composed of three minute and rounded knobs that are arranged forming a triangle on the tooth margin (Fig. 8B).

#### Cervical vertebrae

These elements are characterized by deeply opisthocoelous centra, wide and extensive neural arches, and large and elongate postzygapophyseal processes (Fig. 9A–9C). In contrast to the strongly convex cranial articular surface, the lateral and ventral surfaces, as well as the dorsal surface that forms the floor of the neural canal, are concave. Both the cranial and caudal surfaces of cervical centra show heart-shaped profiles. The prezygapophyses are flat and oval facets lying lateral to the neural arch. The parapophyses consist of oval facets located on the lateral sides of the centra. Each postzygapophyseal process projects caudolaterally and slightly dorsally from the laterodorsal surface of the neural arch. Proximally, each postzygapophyseal process is mediolaterally compressed; further distally, the process gradually becomes mediolaterally expanded to form an craniocaudally elongate postzygapophysis. Caudally along the cervical series, vertebrae show progressively cranioporteriorly shorter centra and postzygapophyseal processes, narrower neural arches, and diapophyses becoming more dorsally oriented.

Cervical ribs are triradiate elements. In the few preserved ribs, the capitulum is over twice as long as the tuberculum and projects laterodorsally and slightly cranially from the craniodorsal region of the rib to articulate with the diapophysis of the centrum. In contrast, the tuberculum extends a short distance laterally and slightly ventrally to meet the parapophysis of the cervical centrum. The shaft of cervical ribs is mediolaterally compressed, with convex lateral and concave medial surfaces, and wedges caudally to a sharp point.

#### Dorsal vertebrae

The morphology of the most cranial dorsal vertebrae (i.e., those articulating with the cranial-most dorsal ribs; Fig. 9D and 9E) is reminiscent of that of the cervical series: the centra are strongly opisthocoelous, with prominently convex cranial articular surfaces, and the postzygapophyseal processes are still present and caudodorsally projected. Unlike cervicals, however, the dorsal region of the neural arch extends further dorsally and slightly caudally in the cranial-most dorsals, and the transverse processes are longer and more dorsally oriented, supporting larger and more elliptical prezygapophyses. More caudal cranial dorsals have substantially elongated centra that are twice as long as they are deep. The ventral surfaces of their centra become keel-shaped and their lateral sides are deeply concave craniocaudally. The transverse processes of these more caudal vertebrae within the cranial dorsal series increase in length, extending dorsolaterally and slightly caudally. The caudal surfaces of the proximal segments of these processes contain deep and oval depressions, just dorsal to the neural arches. The elliptical prezyhapophyses are found above the dorsal margin of the neural arch and face medially and slightly dorsally. The neural spines of the more caudal of the cranial dorsals are very elongated, being at least four times taller than the caudal facet of their centra (Fig. 9F). The neural arches of these vertebrae are also proportionately high.

Middle and caudal dorsal vertebrae (Fig. 9G and 9H) show progressively more craniocaudally compressed centra. The neural canal becomes gradually smaller in diameter than in the cranial dorsals. The transverse processes become laterally directed. The prezygapohyses are wider and more oval in shape, facing mediodorsally. The postzygapophyses are large subelliptical facets that face medioventrally above the caudal margins of the neural arches. No neural spine is completely preserved in any of the partially known dorsal vertebrae. The preserved fragments of dorsal neural spines are thick and elongate, with subrectangular cross-sections. Given the tall nature of the more caudal cranial dorsal, sacral, and caudal neural spines (see below), it is likely that the actual height of the neural spines in the caudal dorsal vertebrae was equally important. Among hadrosaurids, greatly elongate neural spines in dorsal vertebrae are also present in species of *Hypacrosaurus*, which show neural spines that are at least five times the height of their respective centra [43].

#### Sacral vertebrae

The sacrum of *Magnapaulia laticaudus* has particularly tall neural spines (Fig. 10). In the most complete specimen, LACM 17715, the longest (yet not entirely preserved) neural spine is slightly more than four times taller than the cranial surface of the only preserved centrum. This condition is comparable to the elongate neural spines in the sacral and, to a lesser extent, proximal-most caudal vertebrae of the saurolophine hadrosaurid *Barsboldia sicinskii* [44]. Co-ossified sacral centra in *M. laticaudus* show flattened ventral surfaces and craniocaudally concave lateral surfaces. The neural canal is triangular and proportionately large. The transverse processes are massive and project laterally at the base of the neural spines. Thick and triangular laminae connect the base of the transverse processes with the sacral ribs. Those laminae have deeply concave cranial surfaces. As it commonly occurs in hadrosaurids, the sacral ribs also show extensive laminae that fuse to the lateral surface of the centra, to the thick laminae that lie ventral to the transverse processes, and to a robust yoke-like lateral bar that connects to various of the co-ossified vertebrae of the sacrum. The yoke-like bar shows a pitted and flat lateroventral surface for articulation with the medial surface of the ilium.

#### Caudal vertebrae and haemal arches

As noted by Morris [7], the caudal vertebrae of *Magnapaulia laticaudus* are distinctive in having remarkably long neural spines and haemal arches (Fig. 11). Specifically, and continuing the trend observed in the dorsal and sacral vertebrae, both the neural spines and haemal arches are four times longer than the height of their respective centra (e.g., LACM 17705 and 17702; Fig. 11). The caudodorsal orientation of the neural spines and haemal arches become progressively more caudally and less dorsally inclined towards the distal end of the tail. At the same time, the centra change from being shorter proximally to progressively longer distally. Only the proximal segments of the transverse processes are preserved. These extend laterally from the dorsal margin of the lateral sides of the centra. The neural arches are mediolaterally thick and bound relatively narrow neural canals. Dorsal to the neural canal of each vertebra, the bases of the two prezygapophyses fuse craniomedially forming a deep bowl-shaped surface (Fig. 12). The latter is craniodorsally oriented and is bisected by a low sagittal ridge. Dorsally, the bowl-shaped surface is continuous with a wide sulcus (Fig. 12B) that extends a short distance on the cranial surface of the neural spine. The bowl-shaped surface and the sulcus are, to our knowledge, only present in *M. laticaudus* and are regarded here autapomorphic for this taxon. The mediolateral width of the sulcus, which ventrally occupies the entire cranial surface of the base of the neural spine, gradually decreases dorsally until it disappears. Distally along the tail, the bowl-shaped surface becomes shallower. The postzygapophyses are relatively small facets that face lateroventrally and are located on the caudal surface of the base of each neural spine.

#### Sternum

The sternum is a paddle-shaped element composed of an elongate caudolateral process, a short ventromedial process, and an expanded cranial plate. The sternum of *Magnapaulia laticaudus* is known from a single element missing the craniomedial margin of the cranial plate and most of the caudolateral and ventromedial processes (Fig. 13A). The lateral surface of the cranial plate is gently convex transversely and concave longitudinally. In contrast, the medial surface is flat, a condition that extends into the proximal region of the caudolateral process. Cranially, the plate thickens substantially, particularly at its craniodorsal region. The proximal region of the caudolateral process is as compressed as the cranial plate with which it is continuous, showing an elliptical cross-section.

#### Scapula

The scapula is known from a single element preserving the proximal region and the cranial half of the distal blade (Fig. 13E). The cranial margin of the proximal region of the scapula is mediolaterally expanded to form the coracoid and the humeral (glenoid) facets. The coracoid facet is oval and gently concave, being the thickest region of the scapula. As in other lambeosaurines like *Parasaurolophus walkeri* (e.g., ROM 768), *Olorotitan arharensis* (e.g., AEHM 2/845), *Lambeosaurus lambei* (ROM 1218), or *Corythosaurus intermedius* (e.g., CMN 8704), the cranial extension of the craniodorsal region of the scapula bearing the coracoid facet is moderate; the ratio between the distance from the coracoid articulation and the cranial end of the acromial process, and the height between this and the ventral apex of the glenoid facet is 0.4. The glenoid facet is both narrower and longer than the coracoid facet. It is subrectangular in articular view and expanded ventrally. The ventromedial corner of the glenoid facet ends in a prominent apex (Fig. 13E). The acromial process forms a prominent and thick ledge that extends laterally and slightly craniodorsally from the dorsal margin of the proximal scapular region. Below the acromial process, the glenoid fossa occupies most of the lateral surface of the proximal region of the scapula. The proximal constriction is relatively narrow, being 60% of the maximum depth of the articular region of the scapula (depth measured as the distance from the dorsal margin of the acromial process to the apex of the ventral projection of the glenoid).

Most of the ventral and part of the dorsal edges of the scapular blade are eroded. Caudal to the acromial process, the lateral surface of the proximal region of the scapular blade gradually bulges laterally forming the deltoid ridge (Fig. 13E). The latter is poorly developed in *Magnapaulia laticaudus*, consisting of a gentle and longitudinal convexity that crosses caudoventrally the cranial half of the scapular blade. A similarly poorly developed deltoid ridge is typically observed in lambeosaurines, except *Parasaurolophus* spp., and contrasts with the more convex and demarcated ridge of most saurolophine hadrosaurids [41]. The preserved segment of the distal blade of the scapula is strongly curved caudoventrally relative to the horizontal long axis of the acromial process.

#### Coracoid

The coracoid (Fig. 13B–13D) is twice as long dorsoventrally than it is wide craniocaudally. Dorsally, the coracoid is mediolaterally expanded to form the glenoid and scapular facets; ventrally, it is mediolaterally compressed, containing the bicipital tubecle and the large ventral process. The lateral margins of the scapular and glenoid facets meet dorsally forming an angle of 100 degrees, whereas the medial margins of these facets meet forming a 125-degree angle (Fig. 13C). Among lambeosaurines, a 100-degree angle between the lateral margins of the scapular and glenoid facets is also found in *Olorotitan arharensis* (e.g., AEHM 2/845) and *Hypacrosaurus altispinus* (e.g., CMN 8501). The glenoid is more extensive than the scapular facet. Specifically, the lateral margin of the scapular facet is approximately 75% of the length of the lateral margin of the glenoid facet. Both the glenoid and scapular facets are D-shaped in their articular views (Fig. 13D). Below the articular surfaces for the scapula and humerus, the coracoid is pierced by a large foramen. The medial opening of this foramen lies adjacent to the corner where the medial borders of the glenoid and humeral facets meet, whereas the lateral opening is located at a short distance from the articular margins of these facets.

The bicipital tubercle extends cranioventrally and slightly laterally from the cranial border of the coracoid, below the scapular facet (Fig. 13B and 13C). The distal end of the tubercle is abraded. The laterodorsal surface of the bicipital tubercle is dorsoventrally concave in continuity with the equally concave craniomedial margin of the coracoid. Below the bicipital tubercle extends the ventral process of the coracoid. This process is mediolaterally compressed, hook-shaped, and slightly recurved caudally. The parasagittal plane of the ventral process is medially inclined relative to the long axis of the glenoid facet. Dorsoventrally, the ventral process of *Magnapaulia laticaudus* is 70% as wide as it is craniocaudally broad, as in other lambeosaurines [35]. The distal apex of the ventral process is slightly thicker than the remaining cranial and ventral borders, the thicknesses of which remain practically constant.

#### Humerus

As in other iguanodontians, the humerus of *Magnapaulia laticaudus* is mediolaterally compressed proximally and craniocaudally compressed distally (Fig. 14). The articular head of the humerus is massive and protrudes caudally from the caudolateral corner of the element (Fig. 14B, 14D). The deltopectoral crest consists of a thick and extensive flange that extends laterally from the proximal half of the humeral shaft. In *M. laticaudus* the deltopectoral crest is proportionately long proximodistally, accounting for about 60% of the total length of the element (Fig. 14A). Such an elongate deltopectoral crest is also present in (but is not exclusive to) exemplars from other lambeosaurines like *Corythosaurus intermedius* (e.g., ROM 845), *C. casuarius* (e.g., AMNH 5338), *Hypacrosaurus altispinus* (e.g., CMN 8501), *Olorotitan arharensis* (e.g., AEHM 2/845), *Parasaurolophus walkeri* (e.g., ROM 845), and *Tsintaosaurus spinorhinus* (e.g., IVPP V725). The maximum lateral expansion of the deltopectoral crest occurs across its distal fourth and is slightly over 1.8 times the minimum diameter of the shaft. The medial surface of the deltopectoral crest is gently concave transversely. The cranial margin of the crest gradually thickens toward the angular and prominent laterodistal corner. On the caudal surface of the proximal region of the humerus, the caudal tuberosity for the latissimus dorsi muscle forms a large and prominent rugosity in some specimens, like LACM 17716 (Fig. 14D).

Distal to the deltopectoral crest, the humeral shaft reaches its minimum diameter. Its cross-section is subtriangular. Further distally, the humerus expands mediolaterally to form the distal condyles. As in all hadrosaurids, the lateral (ulnar) condyle is wider and more distally prominent than the radial condyle. The intercondylar surface is depressed in both the cranial and caudal sides of the distal end of the humerus; however, the depression is longer and deeper on the caudal side. The lateral and medial surfaces of the ulnar and radial condyles, respectively, are flattened and slightly concave, particularly that of the ulnar condyle.

#### Ilium

The ilium of *Magnapaulia laticaudus* is represented by a nearly complete right exemplar missing the postacetabular process (Fig. 15). The preacetabular process is massive relative to the size of the iliac plate. The proximal region of the process is extremely deep, accounting for 75% of the total depth of the iliac plate of the ilium (measured from the cranial apex of the pubic process to the dorsal margin of the ilium). The preacetabular process is steeply deflected ventrally, so that its long axis forms a 138-degree angle with the horizontal plane defined by the ischiac and pubic processes.

The central plate of the ilium is relatively deep, with a depth/length ratio of 0.81 (depth measured from the point of greater convexity of the dorsal margin of the ilum to the cranial tip of the pubic process; length measured from the caudal corner of the caudodorsal tuberosity of the ischiac process to the cranial tip of the pubic process). Similarly deep or deeper iliac plates are found in other lambeosaurines like *Tsintaosaurus spinorhinus*, *Parasaurolophus walkeri*, *Olorotitan arharensis*, and species of *Corythosaurus*, *Lambeosaurus*, and *Hypacrosaurus* [41]. Extending lateroventrally from the moderately depressed dorsal margin of the iliac plate, the supraacetabular crest is D-shaped and displays a caudally skewed asymmetrical profile in lateral view. The ventral apex of the supraacetabular crest lies craniodorsal to the caudodorsal protuberance of the ischiac process, a condition shared by all hadrosaurids except *Hadrosaurus foulkii* [45]. The ventral projection of the supraacetabular crest is relatively limited, reaching less than 50% of the depth of the iliac plate of the ilium (Fig. 15A). This condition is uncommon in hadrosaurids, where in most taxa the supraacetabular crest projects well below the mid-depth of the ilium. Among lambeosaurines, such a shallow supraacetabular crest is also present in *Amurosaurus riabinini* [42], *Sahaliyania elunchunorum* [40], and *Velafrons coahuilensis* [8], [35]. In *M. laticaudus*, the craniocaudal breadth of the supraacetabular crest is only 46% of the craniocaudal length of the iliac plate; similarly narrow crests occur in many lambeosaurines, like *Amurosaurus riabinini*, *Corythosaurus casuarius*, *Olorotitan arharensis*, or *Hypacrosaurus altispinus* [35].

The pubic process constitutes the cranioventral corner of the iliac plate and the cranial border of the acetabular margin. Like in all hadrosaurids, this process is triangular, relatively short, and ends cranioventrally in a well-defined apex. A prominent and thick ridge borders the lateral surface of the ventral margin of the process. At the other end of the arcuate acetabular margin of the ilium lies the ischiac process. As in all hadrosaurids and some basal hadrosauroids like *Gilmoreosaurus mongoliensis* [46], this process consists of two protrusions. The more cranial of these protrusions expands ventrally and forms the caudal boundary of the acetabular margin. The other protrusion forms an oblique tuberosity that is very prominent laterally and occurs caudodorsal to the acetabular margin.

Only the proximal-most region of the postacetabular process is preserved. The dorsal and ventral margins of this portion of the process are nearly parallel, and oriented caudally and slightly dorsally. The maximum depth of that region of the postacetabular process occurs proximally and is slightly less than half the depth of the central iliac plate. The postacetabular process is slightly inclined medially in relation to the parasagittal orientation of the central plate of the ilium (Fig. 15B).

#### Pubis

The pubis is composed of a large, sheet-like and cranially directed prepubic processs, and a proximal acetabular region containing the iliac process dorsally and the ischiac and postpubic processes ventrally (Fig. 16). As in other lambeosaurines [41], the dorsodistal region of the prepubic process of *M. laticaudus* is further expanded than its ventrodistal region (Fig. 16C). Within Lambeosaurinae, the geometry of the prepubic process is most similar to that of *Hypacrosaurus stebingeri* (e.g., MOR 549), *Parasaurolophus cyrtocristatus* (e.g., FMNH P27393), and *Tsintaosaurus spinorhinus* (e.g., IVPP V728 in [47]) in showing a fist-like lateral profile (Fig. 16C and 16E). This characteristic profile results from the horizontal dorsodistal edge and subrectangular proportions of the distal expansion of the prepubic process, as well as from the deeper and craniocaudally shorter proximal constriction of this process. In contrast, in other lambeosaurines like *Corythosaurus* (e.g., *C. casuarius* AMNH 5338; *C. intermedius* ROM 845) and *Lambeosaurus* (e.g. *L. lambei* TMP 82.38.1; *L. magnicristatus* TMP 66.4.1) the dorsodistal margin of the prepubic process ranges from more rounded to straight and ventrally inclined; the proximal constriction is shallower and proportionately more elongate craniocaudally, and the entire dorsal margin of the prepubic process shows a widely arcuate, shallower concave profile.

The iliac process is tetrahedral and mediolaterally expanded dorsally, as typically seen in other hadrosaurids [48]. The parasagittal plane of the iliac process is laterally deflected in relation to the plane containing the prepubic process, so that the acetabular surface faces caudolaterally (Fig. 16A). That surface is triangular and carved with coarse vertical striations. The ischiac process lies ventral to the iliac process at the other end of the acetabular margin of the pubis. This process is relatively short, subrectangular in lateral view, and projects caudally from the caudoventral end of the proximal region of the pubis (Fig. 16B). Proximally, the ischiac process is greatly compressed mediolaterally (Fig. 16D); in contrast, the articular surface is expanded and subtriangular in distal view (Fig. 16F). The lateral surface of the ischiac process bears a prominent protuberance at its proximal end, where this process converges with the proximal end of the postpubic process, (Fig. 16B). Finally, ventral to the ischiac process there is a long and slender postpubic process. This process is a rod-like shaft that projects caudoventrally, being as long as the combined length of the remaining regions of the pubis (Fig. 16E).

#### Ischium

The ischium of *Magnapaulia laticaudus* consists of a robust proximal region and a long shaft ending distally in a well developed foot-like process (Fig. 17). In the largest and most completely preserved exemplar, LACM 20874, the entire proximal region and shaft of the ischium are massive and extremely thick mediolaterally (Fig. 18). The iliac process projects craniodorsally and its caudodorsal margin (below the distal articular end) forms a 130-degree angle with the dorsal margin of the proximal-most segment of the shaft. As in other lambeosaurine hadrosaurids [48], the caudodorsal region of the iliac process curves caudally, exhibiting a thumb-shaped lateral profile. The caudodorsal end of this curved iliac process is dorsoventrally compressed and as long as in other lambeosaurines like *Lambeosaurus lambei* (e.g., ROM 1218) or *Parasaurolophus cyrtocristatus* (e.g., FMNH P27393). The distal articular surface of the iliac process is greatly expanded mediolaterally and twice as long as it is wide (Fig. 18B, 18D). Most of the mediolateral expansion of the iliac process occurs medial to the parasagittal plane of the ischium and the entire process is slightly deflected medially relative to that plane. The lateral margin of the articular surface is carved with several perpendicularly oriented and short indentations. Adjacent to the cranial edge of the iliac process, the acetabular surface bears a deep sulcus (Fig. 18C, 18D, and 18G). This acetabular sulcus is not usually well preserved in other hadrosaurid ischia but it is, however, likely present in all hadrosaurids (e.g., *Hypacrosaurus altispinus* CMN 8501) as well as in non-hadrosaurid iguanodontians (e.g., *Mantellisaurus atherfieldensis* NHM R11521). Except in LACM 17715 (see below), the acetabular border of the ischium is dorsoventrally narrow, comprising one third of the total dorsoventral breadth of the cranial margin of the proximal region of the ischium. When completely preserved, the acetabular margin of the iliac process forms a 105-degree angle with the lateral margin of the distal articular surface.

The pubic process is very deep, mediolaterally compressed, and displays a subsquared lateral profile (Fig. 18D). The process gradually becomes slightly more expanded dorsally, toward the ventral half of the acetabular margin. In that area, and as in other taxa such as *Corythosaurus intermedius* (e.g., ROM 845), the pubic process contains an oblique flange that borders laterally a flat triangular facet. In general, this facet is rarely readily observable, if preserved at all, in hadrosaurid ischia. The facet faces craniodorsally and forms a 140-degree angle with the vertical cranial margin of the pubic peduncle.

Caudal and slightly ventral to the pubic peduncle, the bone surface is deeply concave and shows a large obturator foramen. This foramen is bounded by a rugose bony rim, the caudal extent of which is thicker and projected caudally. The caudomedial margin of this rim shows a sharp ridge that extends caudodorsally into the medial surface of the proximal segment of the ischiac shaft. The lateral surface of the proximal region of the ischium shows a thick, low longitudinal ridge just above the obturator foramen (Fig. 18H). This ridge extends caudally into the proximal region of the ischiac shaft, where it disappears merging with the lateral surface of the shaft.

The morphology of the proximal region of the ischium of LACM 17715 (Fig. 17A) appears to differ from that in other specimens of *Magnapaulia laticaudus*, such as LACM 20874 (Fig. 18). As preserved, the articular surface of the iliac process in LACM 17715 forms a 135-degree angle with the acetabular margin, an angle that is substantially greater than in LACM 17708 or 20874. Related to the large value of this angle, the lateral profile of the acetabular margin in LACM 17715 is wider than in the other ischia referred to *M. laticaudus*. Likewise, the acetabular sulcus of the iliac process of LACM 17715 is shallower and poorly defined, in contrast to the deep and sharply defined sulcus present in LACM 20874. The dorsal flange and craniodorsal facet of the pubic process of LACM 17715 is also poorly defined and substantially narrower mediolaterally than in LACM 20874. These differences are here attributed to severe erosion of the bone surface in LACM 17715. The entire articular surface, caudal extension, and lateral and medial margins of the iliac process show evidence of substantial abrasion. The bone surface of the acetabular sulcus of the iliac process is heavily abraded, so much that in a few spots the spongy bone is visible. The craniomedial margin of the process has also been eroded, with the consequent loss of the craniomedial corner of the process that is so prominent in LACM 20874. The erosion of this corner in LACM 17715 results in the wider arcuate profile of the acetabular margin of the entire proximal region in this ischium. Erosion extends also onto the pubic peduncle of LACM 17715, particularly along the dorsal margin of the dorsal flange and the cranial and ventral borders of the process. Additionally, the extremely thick nature of the LACM 20874 ischia may be an allometric change during growth of the ischium in this species; indeed, LACM 20874 is substantially greater in size than LACM 17715 and most known hadrosaurid ischia.

The ischiac shaft of *Magnapaulia laticaudus* has an elliptical cross-section throughout most of its length. The long axis of this cross-sectional contour is mediodorsally oriented. Distally, the shaft becomes gradually more compressed mediolaterally. The medial articular surface progressively flattens throughout most of the length of the ischium, particularly at the distal end where it is entirely flat. The distal foot-like process of the ischium is dorsoventrally deep; only the most ventral extent curves antroventrally (Fig. 17A). This morphology is similar to that present in the distal processes of the ischia of many lambeosaurines like *Lambeosaurus lambei* (e.g., ROM 1218) and *Hypacrosaurus altispinus* (e.g., CMN 8501), but it is unlike the relatively slender and longer distal processes of *Parasaurolophus cyrtocristatus* (e.g., FMNH P27393) and *Tsintaosaurus spinorhinus* (e.g., IVPP V725). The ventral edge of the distal end of the ischium is sharply defined and shows a strongly concave lateral profile.

#### Femur

The femur is proportionately slender, due to its relatively elongate shaft (Fig. 19A–19D). Specifically, the total length of the femur is slightly more than nine times greater than the craniocaudal width at mid-shaft. At the proximal end, the extensive greater trochanter is greatly compressed mediolaterally. A wide constriction separates the medially projected femoral head from the proximal end of the femur. The fin-shaped fourth trochanter is relatively long, occupying 30% of the length of the femur. A deep longitudinal sulcus bounds the base of the fourth trochanter and extends further distally along the caudolateral margin of the femur. The distal segment of the shaft is craniocaudally thinnest just proximal to the distal condyles. The cranial intercondylar groove is closed distally by fusion of the craniomedial surface of the distal condyles, as in other lambeosaurines like *Amurosaurus riabinini* [42]. In contrast, the caudal intercondylar groove is not entirely closed, and a very narrow space separates the caudomedial surface of each distal condyle.

#### Tibia

The tibia (Fig. 19E–19H) is composed of a subcylindrical shaft that is craniocaudally and mediolaterally expanded proximally and distally, respectively. Like the femur, the tibia of *M. laticaudus* is proportionately slender; for example, the total length of LACM 17711 is 10 times greater that the mid-shaft diameter. At the proximal end, the two caudolateral condyles are mediolaterally short, craniocaudally wide, and laterally directed. The cnemial crest is very thick and extensive, and its proximomedial corner is slightly curved caudally. The mediolateral breadth of the distal end of the tibia is three times that of the mid-shaft diameter.

#### Fibula

The fibula (Fig. 20A–20C) is long and proportionately slender (total length slightly over 20 times the mid-shaft diameter in LACM 17717). The proximal half of the fibula is mediolaterally compressed, with a lateral surface that is transversely convex and a gently concave medial side. The fibula widens craniocaudally toward the proximal end. This increase in width is very gradual throughout most of the length of the shaft, except along the most proximal segment, where the fibula expands abruptly to exhibit cup-shaped medial and lateral profiles. The proximal articular surface of the fibula is mediolaterally compressed and curves to show a crescentic profile in proximal view. The cranial corner of this surface projects into a prominent peg-like process. The medial side of the proximal end of the fibula is carved with wide and evenly spaced indentations. These short indentations are perpendicularly oriented relative to the straight proximal margin. Distally from mid-shaft the bone thickens mediolaterally and the cross-section of the fibula changes from elliptical to subtriangular. However, near the distal end the fibular shaft becomes again mediolaterally compressed. The distal end is expanded craniocaudally and, cranially, it thickens mediolaterally.

#### Metatarsal III

This is a robust but proportionately elongate element (Fig. 20D and 20E). It is 6 times longer than it is wide at mid-shaft and 3.2 times longer than it is mediolaterally wide across the distal articular surface. Metatarsal III is dorsoventrally and mediolaterally expanded proximally and distally, respectively. The proximal articular surface is crescentic, with concave medial and convex lateral margins. That medial margin represents the caudal border of an extensive depression that exists on the medial surface of the metatarsal. The dorsal portion of the proximal surface is obliquely oriented and faces caudomedially, in contrast to a caudally facing ventral region. The dorsal margin of the proximal surface forms a prominent and thick flange. Cranial to this flange, the dorsal surface of the metatarsal is gently concave and slopes cranially as the central third of the shaft becomes dorsoventrally compressed. The ventral surface of the proximal half of metatarsal III is gently convex along its medial border and more prominent medially than laterally. The articular distal surface of the element is slightly saddle-shaped and shows a trapezoidal profile, with a ventral margin that is mediolaterally wider than the dorsal one. The texture of this surface is smooth at the center and carved with transverse indentations at the lateral and medial margins. The medial surface of the distal end is more concave than the lateral surface.

#### Integument

The external morphology of the skin of *Magnapaulia laticaudus* is partially known from various patches with impressions and natural casts of the scales (Fig. 21). According to Morris [7], these patches were found associated to the cranial dorsal vertebrae. They consist of a mosaic of hexagonal to oval tubercles ranging in size from 2.5–3 to nearly ten millimeters in width. The tubercles are tightly arranged and, occasionally, larger oval structures measuring 35 to 40 millimeters in maximum width are found among them (Fig. 21E, arrow). These large structures lack any apparent pattern of distribution within the scalation and may represent osteoderms embedded in the skin. At least one the fossils of these structures preserves impressions of fine radial ridges [7]. The findings of integumentary casts and impressions abound in the fossil record of hadrosaurids [1], [49]–[60]. The size, polygonal shape, and arrangement of the integumentary tubercles observed in *M. laticaudus* does not differ from those found in many other hadrosaurids like lambeosaurines *Corythosaurus casuarius* (e.g., AMNH 5240), *C. intermedius* (e.g., ROM 845), *Lambeosaurus lambei* (e.g., ROM 1218, CMN 351 and 8703), *L. magnicristatus* (e.g., TMP 66.4.1), and *Hypacrosaurus altispinus* (e.g., AMNH 5217), and saurolophines *Brachylophosaurus canadensis* (e.g., MOR 1071), *Gryposaurus notabilis* (e.g., ROM 764), *Edmontosaurus annectens* (e.g., AMNH 5060, SM R4036), *E. regalis* (e.g., CMN 8399), and *Saurolophus angustirostris* (e.g., ZPAL MgD-I 159). Large osteoderm-like structures of similar size, morphology (including the fine radial ridges), and distribution in the skin as those observed in *M. laticaudus* are also known in some lambeosaurines like *Corythosaurus* sp. (e.g., CMN 2226), *C. casuarius* (e.g., AMNH 5240), and *L. lambei* (e.g., CMN 351).

### Phylogenetic position of *Magnapaulia laticaudus*

The phylogenetic analysis returned a single most parsimonious tree of 638 steps (CI = 0.61, RI = 0.77) (Fig. 22). *Magnapaulia laticaudus* was recovered as the sister taxon to the only other known lambeosaurine from México (and southern North America in general), *Velafrons coahuilensis*. Both taxa form a clade of southern North American lambeosaurines and this phylogenetic placement supports removal of the Baja California lambeosaurine from the genus *Lambeosaurus*. The clade is unambiguously supported by an ilium with a supraacetabular crest that extends ventrally more than 25% but less than half the depth of the central iliac plate (a condition convergent in *Olorotitan arharensis*, *Shaliyania elunchunorum*, and basal hadrosauroids *Bactrosaurus johnsoni* and *Gilmoreosaurus mongoliensis*). An ambiguous synapomorphy (character state distribution unknown in *Charonosaurus jiayinensis*, *Parasaurolophus tubicen* and *P. cyrtocristatus*) also unites *V. coahuilensis* and *M. laticaudus*: triangular external naris gradually closing caudodorsally and with a length/width ratio between 1.85 and 2.85 (convergent in *Olorotitan arharensis*). Although the external nares of *Lambeosaurus* spp., *Corythosaurus* spp., and *Hypacrosaurus stebingeri* show a similar contour than those of *M. laticaudus* and *V. coahuilensis*, their length/width ratio are greater than 2.85 ([35]: fig. D.22]).

Additionally, the *Magnapaulia-Velafrons* clade was inferred to be the sister clade to a more speciose monophyletic group of helmet-crested forms that included *Hypacrosaurus* spp., *Olorotitan arahrensis*, *Corythosaurus* spp., and *Lambeosaurus* spp. One unambiguous synapomorphy supported this sister relationship: an angle between the dentary proximal-most edentulous slope and the tooth row of 150 degrees or greater (reversed to a lower than 150-degree angle in *H. stebingeri*).

Three ambiguous synapomorphies, the distribution of which are variably known in *Sahaliyania*, *Tsintaosaurus*, *Pararhabdodon*, *Jaxartosaurus*, and *Aralosaurus*, support inclusion of *M. laticaudus* in the clade integrated by *Hypacrosaurus* spp., *Olorotitan arharensis*, *Corythosaurus* spp., *Lambeosaurus* spp., and the *Charonosaurus*-*Parasaurolophus* spp. subclade: the oral margin of the premaxilla being slightly deflected ventrally and dorsoventrally thicker toward the sagittal plane of the snout; elongation of the medial process of the premaxilla to meet the lateral process and form the caudal margin of the external naris; and absence of premaxillary accessory fossae.

At a more inclusive level, *Magnapaulia laticaudus* shares various synapomorphies with the clade including all lambeosaurines except the more basal forms *Tsintaosaurus*, *Pararhabdodon*, *Jaxartosaurus*, and *Aralosaurus*: faint secondary ridges on the enameled surfaces of dentary tooth crowns; a triangular and craniocaudally abbreviated craniodorsal region of the maxilla; a large maxillary foramen opening on the craniodorsal surface of the maxilla, along the premaxilla-maxilla contact; absence of craniodorsal medial process of the maxilla and presence of craniodorsally-sloping shelf underlying the premaxilla; absence of premaxillary foramen; given the morphology and extent of the premaxilla, possibly the nasal folded and rotated caudodorsally; a coracoid ventral process with a depth/proximal width ratio between 0.65 and 0.80; and the distal blade of the prepubic process of the pubis with a more expanded dorsal region. These synapomorphies are, however, ambiguous since their distribution in all or some of the four basal lambeosaurines noted above are variably unknown.

The topology recovered in the present analysis differs in two important aspects from the tree derived from the only other previous cladistic study that included *Magnapaulia laticaudus* [41]. In the present analysis the Asian *Amurosaurus-Sahaliyania* clade does not form a sister relationship with the *Hypacrosaurus* clade, but was recovered as the sister clade to the major monophyletic group including all lambeosaurines except *Tsintaosaurus*, *Pararhabdodon*, *Jaxartosaurus*, and *Aralosaurus*.

Another remarkable difference concerns the position of *Olorotitan arharensis*, deeply nested here among the helmet-crested lambeosaurines as the sister species to *Hypacrosaurus altispinus*. In the phylogeny by Prieto-Márquez [41], *O. arharensis* was positioned as a relatively basal taxon outside the clade including all lambeosaurines to the exclusion of *Tsintaosaurus*, *Pararhabdodon*, *Jaxartosaurus*, and *Aralosaurus*. However, the phylogenetic position inferred here for *O. arharensis* is more congruent with recent analyses, in which this species appeared forming a polytomy with the genera *Hypacrosaurus* and *Corythosaurus* [36], [40] or as the sister species to a *Corythosaurus casuarius*-*Hypacrosaurus* spp. clade [37]. Here, *Hypacrosaurus altispinus* and *Olorotitan arharensis* are unambiguously united based on the presence in both taxa of a straight, nearly vertical cranial margin of the rostral process of the jugal (convergent in *Tsintaosaurus spinorhinus* and an unnamed hadrosaurid from the late Maastrichtian Tremp Formation of northern Spain [61]), and presence of a brevis shelf-like surface on the postacetabular process of the ilium.

These differences between the topology of Prieto-Márquez [41] and the one herein presented are due to substantial changes in the character-taxon matrix used in this analysis, specifically: 1) inclusion of the character coding of the dentary of *M. laticaudus* (Supporting Information S2); 2) exclusion (see description of the ischium above) of character 264 of Prieto-Márquez [41] from the data matrix; and 3) updating the coding of 18 characters (15 cranial and three appendicular) for *Olorotitan arharensis* (Supporting Information S2 and S3), according to the detailed anatomical description of this taxon and phylogenetic analysis of lambeosaurine relationships by Godefroit et al. [37].

## Discussion

### Comparison with *Velafrons coahuilensis*

Aside from *Magnapaulia laticaudus*, the only other lambeosaurine taxa erected so far from Mexico is *Velafrons coahuilensis*, from the late Campanian Cerro del Pueblo Formation outcropping near the town of Rincón Colorado, state of Coahuila [8]. Although *M. laticaudus* and *V. coahuilensis* share a similar morphology of the external naris, they notably differ in the orientation of the rostroventral process of the maxilla (Table 4). Specifically, whereas the process in *M. laticaudus* is ventrally deflected, that of *V. coahuilensis* is horizontally oriented [8:fig. 5A]. Comparing their premaxillae, the external naris of *V. coahuilensis* is approximately 25 cm in length in the type specimen CPC-59, and 27 cm in LACM 17715. This indicates that CPC-59 and LACM 17715 likely represent similar ontogenetic stages, clearly substantially smaller than the larger individuals known for *M. laticaudus* (e.g., LACM 17712; see Fig. 2). That the external naris morphology does not differ betweem these two specimens is consistent with them being of similar ontogenetic stage and with the observation that the shape of the external naris appears to be influenced by ontogeny [62]. Finally, the autapomorphies of *V. coahuilensis* [8] occur in cranial elements that are not preserved in *M. laticaudus*, such as the postorbital, quadrate, and ceratobranchial (Table 4). Given the existence of taxonomically and phylogenetically informative [35], [41] differences between these two species (Table 4), and the lack of overlapping elements containing diagnostic characters for *V. coahuilensis*, we deemed more appropriate to maintain the taxonomic separation between *Magnapaulia* and *Velafrons*, in agreement with previous studies [8].

**Table 4:** Comparison of selected cranial character states in *Velafrons coahuilensis* and *Magnapaulia laticaudus*.

Character	*Velafrons coahuilensis*	*Magnapaulia laticaudus*
Premaxilla, length/width ratio of external naris	between 1.85 and 2.85	between 1.85 and 2.85
Maxilla, orientation of rostroventral process	horizontal	pendant, ventrally deflected
Maxilla, length of ectopterygoid shelf	over 35% of maxilla length	over 35% of maxilla length
Maxilla, orientation of ectopterygoid shelf	horizontal	horizontal
Quadrate with narrow quadratojugal notch	present	quadrate not preserved
Postorbital with dorsally arching squamosal process	present	postorbital not preserved
Ceratobranchial with rounded rostral end	present	ceratobranchial not preserved
Premaxillonasal fan-like crest	present	crest not preserved
Jugal, relative depth of orbital and infratemporal constrictions	deeper infratemporal constriction	subqually deep
Jugal, depth of caudoventral flange/depth of infratemporal constriction ratio	less than 1.35	less than 1.35
Dentary, origination of the rostral deflection (distance measured taking the caudal margin of the coronoid process as starting point)	between two thirds and 70% of the length of the dental battery	between two thirds and 70% of the length of the dental battery

### Biogeographic hypothesis

**Figure 23:**
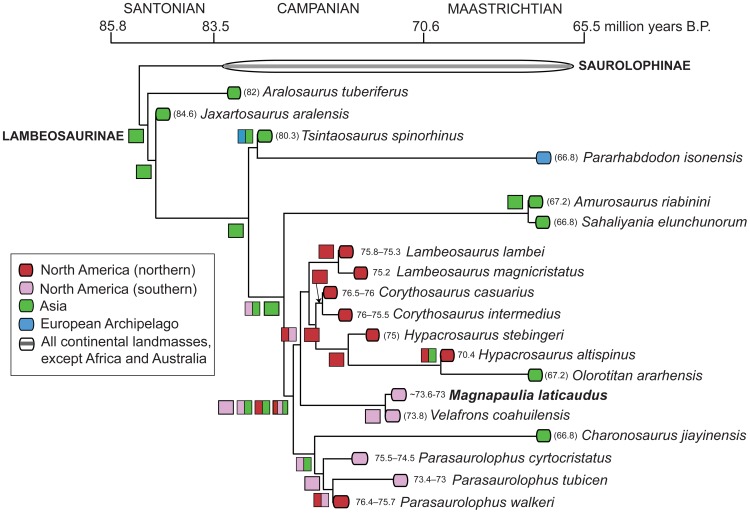
Time-calibrated phylogram of Lambeosaurinae based on the phylogenetic hypothesis shown in Fig. 22. The letters at each node indicate ancestral areas as inferred in the Dispersal-Vicariance analysis; a combination of two letters represents a widespread range of the ancestor in both of the areas indicated by each letter. The numbers to the left of the taxon names are the estimated ages in millions of years. When absolute age estimates are not available but only time ranges (e.g., middle-late Maastrichtian), the age of the taxon is approximated as the mid-point of a particular geochronological stage (numbers between brackets). The literature sources for each of the taxon's ages are as follows: *Amurosaurus riabinini* [42]; *Aralosaurus tuberiferus* [79], [80]; *Charonosaurus jiayinensis* [81]; *Corythosaurus* spp., *Lambeosaurus* spp., and *Parasaurolophus walkeri* [82]; *Hypacrosaurus altispinus* [43]; *H. stebingeri* [5]; *Magnapaulia laticaudus* [21]; *Jaxartosaurus aralensis* [83]; *Olorotitan arharensis* [37]; *Pararhabdodon isonensis* [84]; *Parasaurolophus tubicen* and *P. cyrtocristatus* [10]; *Sahaliyania elunchunorum* [40]; *Tsintaosaurus spinorhinus* [85]; and *Velafrons coahuilensis* [8]. Geochronological ages are from Gradstein et al. [86].

The reconstruction of ancestral areas for the nodes of the phylogenetic hypothesis inferred in this study supports an Asian origin for Lambeosaurinae, as previously proposed by other authors [6], [36], [40], [42], [63], [64] no later than the Santonian (Fig. 23). It is, however, uncertain by which processes and from/to which areas did lambeosaurines extend their ranges into North America. Nevertheless, the results from DIVA show unambiguously that the most parsimonious range of the most recent common ancestor of the northern North American clade of helmet-crested lambeosaurines and the southern *Velafrons-Magnapaulia* clade was widespread throughout North America. The split between the northern and southern clades is explained as the result of vicariance, which is postulated to occur no later than the middle-late Campanian (Fig. 23).

### The status of *Magnapaulia laticaudus* as a ‘giant’ hadrosaurid

**Figure 24:**
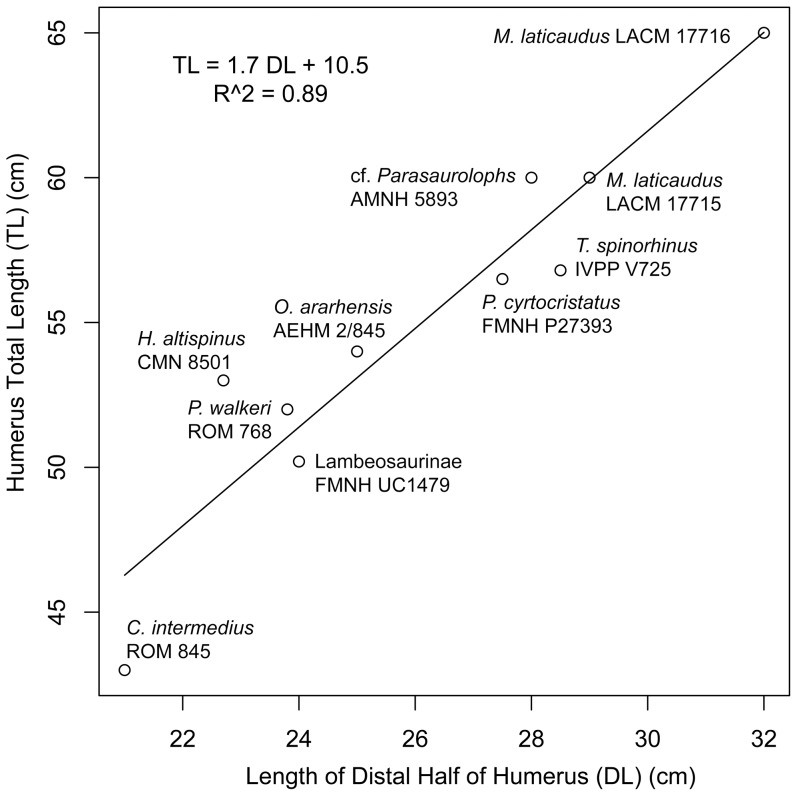
Scatterplot showing the relationship between the distal half of the humerus and the total length of the bone for a sample of lambeosaurine hadrosaurids.

**Figure 25:**
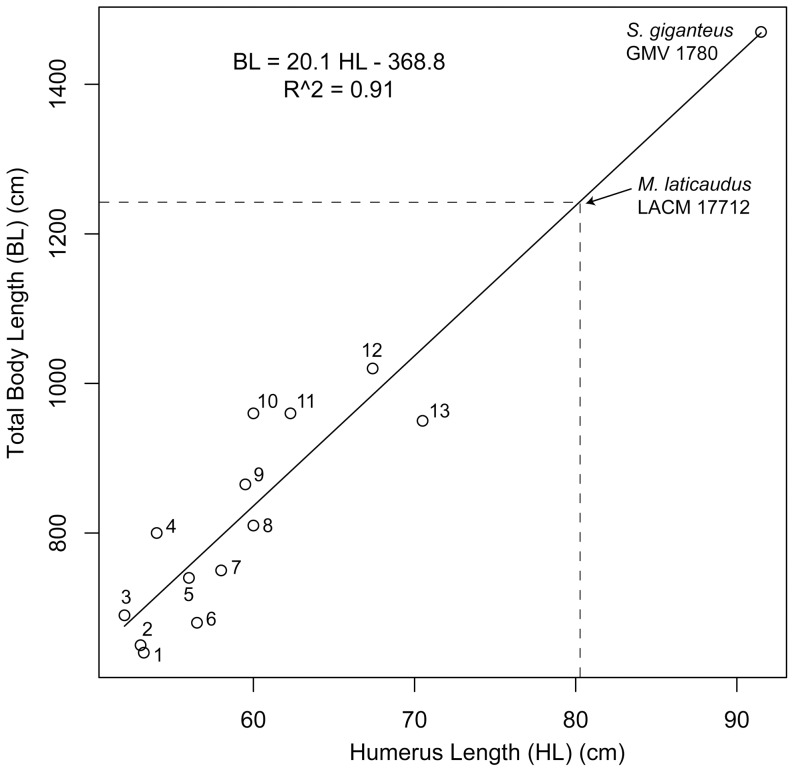
Scatterplot showing the relationship between the length of the humerus and total body length for a sample of hadrosaurid dinosaurs. Lengths measured by the authors, unless otherwise indicated. The numbers indicate the following specimens: 1, *Prosaurolophus maximus* ROM 787; 2, *Hypacrosaurus altispinus* CMN 8501; 3, *Edmontosaurus annectens* SM R4036; 4, *Olorotitan ararhensis* AEHM 2/845 (body length from [37]); 5, *E. annectens* YPM 2182; 6, *Parasaurolophus cyrtocristatus* FMNH P27393; 7, *Corythosaurus casuarius* AMNH 5338; 8, *Gryposaurus notabilis* ROM 764; 9, *Brachylophosaurus canadensis* MOR 794; 10, *Saurolophus osborni* AMNH 5220; 11, *E. regalis* CMN 8399; 12, *E. regalis* ROM 801; *G. notabilis* MSNM V345 (data after [87]). The measurements from *Shantungosaurus giganteus* are from [65].

Adult hadrosaurids (i.e., specimens with over 85% of maximum recorded skull length for the species [43]) typically reached body lengths of 7–10 m [35]. Some bones of *Magnapaulia laticaudus* represent individuals well in excess of 10 m in length. For example, the humerus of LACM 17712 measures 723 mm in length as preserved (the bone lacks its proximal region; Fig. 14E, F). The total length of the humerus of LACM 17712 was estimated in 803 mm by regressing the length of the distal humeral half with the total length of the element, using a sample of lambeosaurine hadrosaurids (Fig. 24). From this estimate, and the relationship between humerus length (Table 5) and total body length in hadrosaurids (Fig. 25), the body length of LACM 17712 was estimated in approximately 12.5 m, a more conservative figure than the 14–15 m considered by Morris [7]. Other exemplars, such as LACM 20874 (Fig. 18), may also represent individuals reaching over 12 m in length. Notably, Morris [25] reported an incomplete lambeosaurine humerus, LACM 26757, from the El Gallo Formation. The proximal end of this humerus was restored and its total length was estimated in 950 mm [25]. We have not been able to locate this humerus in the collections of the LACM nor the IGM, and the bone may or may not belong to *M. laticaudus*. Yet, if Morris' restoration and length estimation are accurate, this element would represent a lambeosaurine nearing 15 m in length, a dimension comparable to that reported for *Shantungosaurus giganteus* [65] (Fig. 25).

**Table 5:** Comparison of humeral lengths (in mm) for a sample of hadrosaurid species.

Taxon	Specimen	Humerus Length
**Saurolophinae**		
*Brachylophosaurus canadensis*	MOR 794	595
*Edmontosaurus regalis*	CMN 8399	623
*Edmontosaurus regalis*	ROM 801	674
*Edmontosaurus annectens*	SM R4036	520
*Edmontosaurus annectens*	YPM 2182	560
*Gryposaurus notabilis*	ROM 764	600
*Gryposaurus notabilis*	MSNM V345	705
*Prosaurolophus maximus*	ROM 787	532
*Saurolophus osborni*	AMNH 5220	600
*Shantungosaurus giganteus*	GMV 1780	915
**Lambeosaurinae**		
*Magnapaulia laticaudus*	LACM 17712	*803
*Magnapaulia laticaudus*	LACM 17716	650
*Magnapaulia laticaudus*	LACM 17715	600
cf *Parasaurolophus*	AMNH 5893	600
*Tsintaosaurus spinorhinus*	IVPP V725	568
*Parasaurolophus cyrtocristatus*	FMNH P27393	565
*Olorotitan arharensis*	AEHM 2/845	540
Lambeosaurinae indeterminate	FMNH UC1479	502
*Parasaurolophus walkeri*	ROM 768	520
*Hypacrosaurus altispinus*	CMN 8501	530
*Corythosaurus casuarius*	ROM 845	430

The asterisk indicates a value estimated using the regression equation shown in Fig. 24.

Overall, the above estimates position *Magnapaulia laticaudus* as the largest recorded lambeosaurine (individuals from the other taxa of the clade do not surpass the 9–10 m in length; Fig. 25) and one of the largest known hadrosaurid species, not only in North America, but in the world. In North America, *M. Laticaudus* might have been rivaled in size by at least some exemplars of *Edmontosaurus annectens* (e.g., NHM 3656, the dentary of which is over 1 m in length) and probably the largest individuals of *Kritosaurus navajovius* [66] and *Anasazisaurus horneri* [67]. In a global context, our more conservative estimates of the body length of *M. laticaudus* set this animal's dimensions below the 14.7 m reported for *Shantungosaurus giganteus* [65], which still remains the largest known described hadrosaurid species.

### Conclusions

A detailed comparative osteology of the available cranial, appendicular, and axial elements of *Magnapaulia laticaudus* leads to a revised diagnosis, phylogenetic position, and biogeographic hypothesis of this taxon. The possession of the autapomorphic elongate haemal arches of proximal caudal vertebrae (being at least four times longer than the depth of their respective centra) and the base of prezygapophyses in caudal vertebrae merging to form a bowl-shaped surface, coupled with the diagnostic combination of pendant cranioventral process of the maxilla, tear-shaped external naris with length/width ratio between 1.85 and 2.85, and neural spines of dorsal, sacral, and proximal caudal vertebrae being at least four times the depth of their respective centra, argues against previous referral of this species to *Lambeosaurus* and supports the erection of a new genus.

*Magnapaulia laticaudus* was parsimoniously inferred to be the sister taxon to *Velafrons coahuilensis*, also from the late Campanian of México. Both species represent a unique clade of southern North American lambeosaurines, which, according to the results of Dispersal-Vicariance analysis, split from the northern forms via vicariance, prior to the middle-late Campanian (∼75 mya), from a common ancestor that lived in both the northern and southern regions of the continent.

## Methods

### Materials

The osteological descriptions and character coding of *Magnapaulia laticaudus* were based on first hand examination of the holotype and referred materials of this species housed at the LACM in Los Angeles (California, USA) and the Instituto de Geología at the Universidad Autónoma Nacional de México (México DF) (Supporting Information S1).

### Morphometric analysis

The morphology of the external naris in the premaxilla of lambeosaurine hadrosaurids was analyzed using its boundary representation by applying the square-root elastic framework for shape analysis of curves described by Joshi et al. [33], [34], [68]. Our goal here was to quantify the dissimilarity of the external nares between the *Magnapaulia laticaudus* and the other lambeosaurine species for which this premaxillary region is preserved. In this method, the shape outlines of the external nares of 30 lambeosaurine specimens were represented by continuous curves and parameterized by their square-root velocity functions. For each shape, the square-root velocity function maps each point on the curve to a vector in R^2^. This function is automatically invariant to translation and is further made invariant to scale by normalizing its magnitude. We analyzed the shapes of curves as elements of an infinite-dimensional, nonlinear manifold equipped with a well-defined Riemannian (i.e., non-Euclidean) metric. Here the geometrical differences between shapes were studied under invariances to rigid rotations, translations, uniform scaling, and reparameterizations of curves. The shape differences were then quantified elastically by computing geodesics (shortest paths) on the shape space, which is an infinite-dimensional sphere where geodesics were analytically specified [33], [34], [68]. We note that this is an improvement over our previous method [69] where we only allowed non-elastic matching of shapes. In practice, our approach began by tracing digital contours of the subject shapes using a Wacom Graphire graphic tablet (Wacom Co., Ltd., Saltama, Japan) with Illustrator CS2 (Adobe Systems Inc., San Jose, CA, USA). The digitized shapes were then saved as svg vector files and imported into the software program MATLAB (The MathWorks, Natick, MA, USA). The tools and algorithms used for analysis were executed within the MATLAB programming environment. Upon completion of the shape analysis, we obtained a dissimilarity matrix of pairwise geodesic distances among all the narial shapes. These results were summarized and visualized graphically via non-metric multidimensional scaling, a technique that transforms a multidimensional, non-Euclidean space into a coordinate Euclidean space with a limited number of dimensions [70].

### Phylogenetic inference

The phylogenetic position of *Magnapaulia laticaudus* within Lambeosaurinae was inferred via maximum parsimony analysis. We used the character matrix of Prieto-Márquez [41] that consists of 286 equally weighted morphological characters (196 cranial and 90 postcranial). All the characters of that matrix were included, except character 264 (angle between the lateral articular margin of the iliac process of the ischium and the acetabular margin of this process). This character was excluded because the angle it is based upon is highly susceptible of varying due to abrasion (see description of the ischium above). Counting *M. laticaudus*, the present analysis included 30 hadrosauroid species, consisting of 22 Hadrosauridae (of which 19 species belong to Lambeosaurinae) and eight non-hadrosaurid Hadrosauroidea, which provide a thorough representation of the known diversity of hadrosaurids. The non-hadrosauroid iguanodontian *Iguanodon bernissartensis* was selected as outgroup taxon to hadrosauroids. The character coding of *Olorotitan arharensis* was updated following to the revised descriptions and character coding provided by Godefroit et al. [37] (see Supporting Information S2 and S3). The search for the optimal tree(s) was conducted in TNT version 1.0 [71]. A heuristic search of 10,000 replicates using random additional sequences was performed, followed by branch swapping by tree-bisection-reconnection holding 10 trees per replicate. Bremer support [72] was assessed by computing decay indices [73] using TNT. Bootstrap proportions [74] were calculated with PAUP* version 4.0b10 [75]. We used 5,000-replicate heuristic searches, where each search was conducted using random additional sequences with branch-swapping by subtree pruning and regrafting at 25 replicates. Phylogenetic nomenclature followed definitions and usage in Prieto-Márquez [41].

### Ancestral areas

Finally, we implemented Dispersal-Vicariance Analysis (or DIVA [76], [77]) to the reconstruction of the ancestral areas for all internal nodes of the phylogeny resulting from parsimony analysis. DIVA assumes allopatric speciation as a result of vicariance as a null hypothesis, and also considers dispersal, extinction, and duplication as alternative hypotheses to explain the observed distribution of taxa. DIVA uses a model in which vicariance, sympatric speciation, dispersal, and extinction are given costs that are related to the likelihood of occurrence of these events [78]. Thus, vicariance and sympatric speciation receive a cost of zero, whereas dispersal and extinction have a cost of one per area unit added or deleted from the distribution [77]. The method was implemented in the program DIVA 1.1 [76] using the optimization algorithm of Ronquist [77]. It uses parsimony as optimality criterion, searching for the reconstruction that minimizes the number of dispersal and extinction events required to explain the geographical distribution of terminal taxa. In the present analysis, we considered only four large continental areas, Europe, Asia, and northern and southern North America, which contain the fossil record of all taxa under consideration in our phylogenetic and ancestral range reconstruction analyses.

### Nomenclatural acts

The electronic version of this document does not represent a published work according to the International Code of Zoological Nomenclature (ICZN), and hence the nomenclatural acts contained in the electronic version are not available under that Code from the electronic edition. Therefore, a separate edition of this document was produced by a method that assures numerous identical and durable copies, and those copies were simultaneously obtainable (from the publication date noted on the first page of this article) for the purpose of providing a public and permanent scientific record, in accordance with Article 8.1 of the Code. The separate print-only edition is available on request from PLoS by sending a request to PLoS ONE, 1160 Battery Street, Suite 100, San Francisco, CA 94111, USA along with a check for $10 (to cover printing and postage) payable to “Public Library of Science”.

In addition, this published work and the nomenclatural acts it contains have been registered in ZooBank, the proposed online registration system for the ICZN. The ZooBank LSIDs (Life Science Identifiers) can be resolved and the associated information viewed through any standard web browser by appending the LSID to the prefix “http://zoobank.org/”. The LSID for this publication is: urn:lsid:zoobank.org:pub:B71CDFFA-9B8D-4A77-B068-A11D444D5171. The article is deposited in the PubMedCentral and LOCKSS digital archives.

## Supporting Information

Supporting Information S1 List of hadrosaurid materials recovered from Upper Cretaceous strata of Baja California.
(XLS)

Supporting Information S2 Character state codings for *Magnapaulia laticaudus* and *Olorotitan ararhensis* for the 255 morphological characters included in the phylogenetic analysis.
(XLS)

Supporting Information S3 Revised codings for *Olorotitan ararhensis* for 18 of the 255 morphological characters included in the phylogenetic analysis.
(XLS)
